# Conformational dynamics in the disordered region of human CPEB3 linked to memory consolidation

**DOI:** 10.1186/s12915-022-01310-6

**Published:** 2022-06-03

**Authors:** D. Ramírez de Mingo, D. Pantoja-Uceda, R. Hervás, M. Carrión-Vázquez, D. V. Laurents

**Affiliations:** 1 Instituto Cajal, IC-CSIC, Avda. Doctor Arce 37, 28002 Madrid, Spain; 2 Instituto de Química-Física Rocasolano, IQFR-CSIC, Serrano 119, 28006 Madrid, Spain; 3 School of Biomedical Sciences, The University of Hong Kong, Pokfulam, Hong Kong, China

**Keywords:** Memory consolidation, Intrinsically disordered proteins, NMR spectroscopy

## Abstract

**Background:**

Current understanding of the molecular basis of memory consolidation points to an important function of amyloid formation by neuronal-specific isoforms of the cytoplasmic polyadenylation element binding (CPEB) protein family. In particular, CPEB is thought to promote memory persistence through formation of self-sustaining prion-like amyloid assemblies at synapses, mediated by its intrinsically disordered region (IDR) and leading to permanent physical alterations at the basis of memory persistence. Although the molecular mechanisms by which amyloid formation takes place in CPEB have been described in invertebrates, the way amyloid formation occurs in the human homolog CPEB3 (hCPEB3) remains unclear. Here, we characterize by NMR spectroscopy the atomic level conformation and ps-ms dynamics of the 426-residue IDR of hCPEB3, which has been associated with episodic memory in humans.

**Results:**

We show that the 426-residue N-terminal region of hCPEB3 is a dynamic, intrinsically disordered region (IDR) which lacks stable folded structures. The first 29 residues, M_1_QDDLLMDKSKTQPQPQQQQRQQQQPQP_29_, adopt a helical + disordered motif, and residues 86–93: P_83_QQPPPP_93_, and 166–175: P_166_PPPAPAPQP_175_ form polyproline II (PPII) helices. The (VG)_5_ repeat motif is completely disordered, and residues 200–250 adopt three partially populated α-helices. Residues 345–355, which comprise the nuclear localization signal (NLS), form a modestly populated α-helix which may mediate STAT5B binding. These findings allow us to suggest a model for nascent hCPEB3 structural transitions at single residue resolution, advancing that amyloid breaker residues, like proline, are a key difference between functional versus pathological amyloids.

**Conclusion:**

Our NMR spectroscopic analysis of hCPEB3 provides insights into the first structural transitions involved in protein–protein and protein-mRNA interactions. The atomic level understanding of these structural transitions involved in hCPEB3 aggregation is a key first step toward understanding memory persistence in humans, as well as sequence features that differentiate beneficial amyloids from pathological ones.

**Areas:**

Biophysics, Structural Biology, Biochemistry & Neurosciences.

**Supplementary Information:**

The online version contains supplementary material available at 10.1186/s12915-022-01310-6.

## Background

The molecular basis of long-term memory, which endures decades despite being built by ephemeral biomolecules, has long fascinated biochemists [1]. Seminal findings by Si, Lindquist, and Kandel suggested that long-term changes in synaptic efficacy require a self-perpetuating amyloid state in the *Aplysia* CPEB (*Ap*CPEB) [2, 3]. This change in CPEB’s conformation would lead to permanent alterations at the synapse constituting a physical substrate of memory persistence. To attain the aggregated state necessary to stabilize memory, *Ap*CPEB contains an N-terminal IDR that is very rich in glutamine (Q) residues which losses α-helix and gains coiled-coil and β-sheet structure during amyloid formation *in vitro* [4, 5].

The *Drosophila* homolog of CPEB, called Orb2, behaves in a similar fashion even though its N-terminal IDR has a lower glutamine residue content and a more tightly regulated amyloid formation [6–9]. Indeed, inhibition of Orb2 amyloid formation targeting the N-terminal IDR specifically impairs memory consolidation, but not short-term memory in *Drosophila* [6, 10]. Due to numerous histidine (H) residues in the Orb2 Q/H-rich amyloid core comprised in the N-terminal IDR, pH may regulate this structure’s stability as suggested by CryoEM analysis [5] and characterization by NMR spectroscopy [11]. In mammals, the N-terminal IDR of the neuronal-specific isoform of CPEB3 is crucial for amyloid formation and memory consolidation [12, 13]. The regulation of functional amyloid formation in mammalian CPEB3 appears to be even more sophisticated due to multiple mechanisms involving post-translational modifications [14] and feedback loops to maintain hCPEB3 expression levels [13]. Compared to the *Aplysia* and *Drosophila* homologs, hCPEB3’s content of glutamine residues in its 426-residues long IDR is lower, and it contains diverse segments which are enriched for certain residues such as Ser, Ala, Pro, Gly + Val, and hydrophobic residues (Table 1).

**Table 1:**
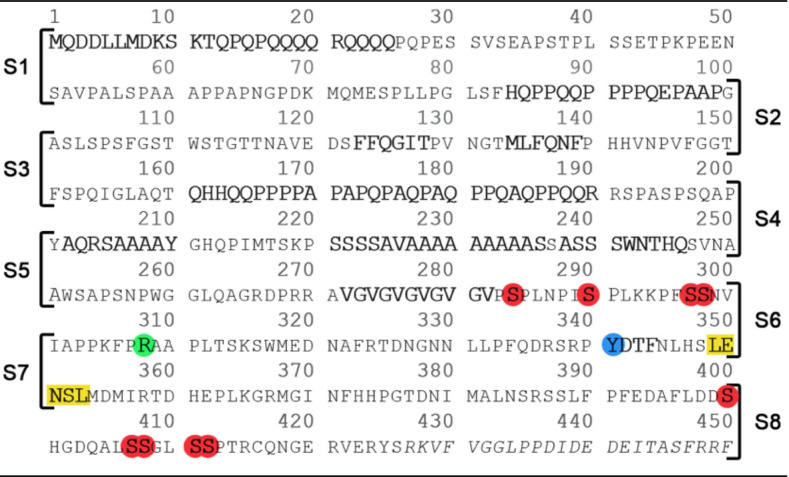
Sequence of the disordered N-terminal region of hCPEB3

Segment 1 spans hCPEB3 residues 1-100; segment 2, residues 51 – 150; segment 3, residues 101 – 200; segment 4, residues 151 – 250; segment 5, residues 201 – 300; segment 6, residues 251 350; segment 7, residues 302 – 400 and finally segment 8, which is composed of residues 352 – 450. Residues of structural or functional interest are in bold font. The putative dimethyl-Arg site (R308) is highlighted in green. The putative phosphoTyr site, Y341 plausibly recognized by STAT5B’s SH2 domain is highlighted in blue. The predicted nuclear export signal, L349-L353, is highlighted in yellow. Putative phosphorylation sites: S284, S290, S297, S298, S400, S407, S408, S411 and S412 are highlighted in red. Finally, residues belonging to the first RRM1 domain are written in italics.

**Fig. 1:**
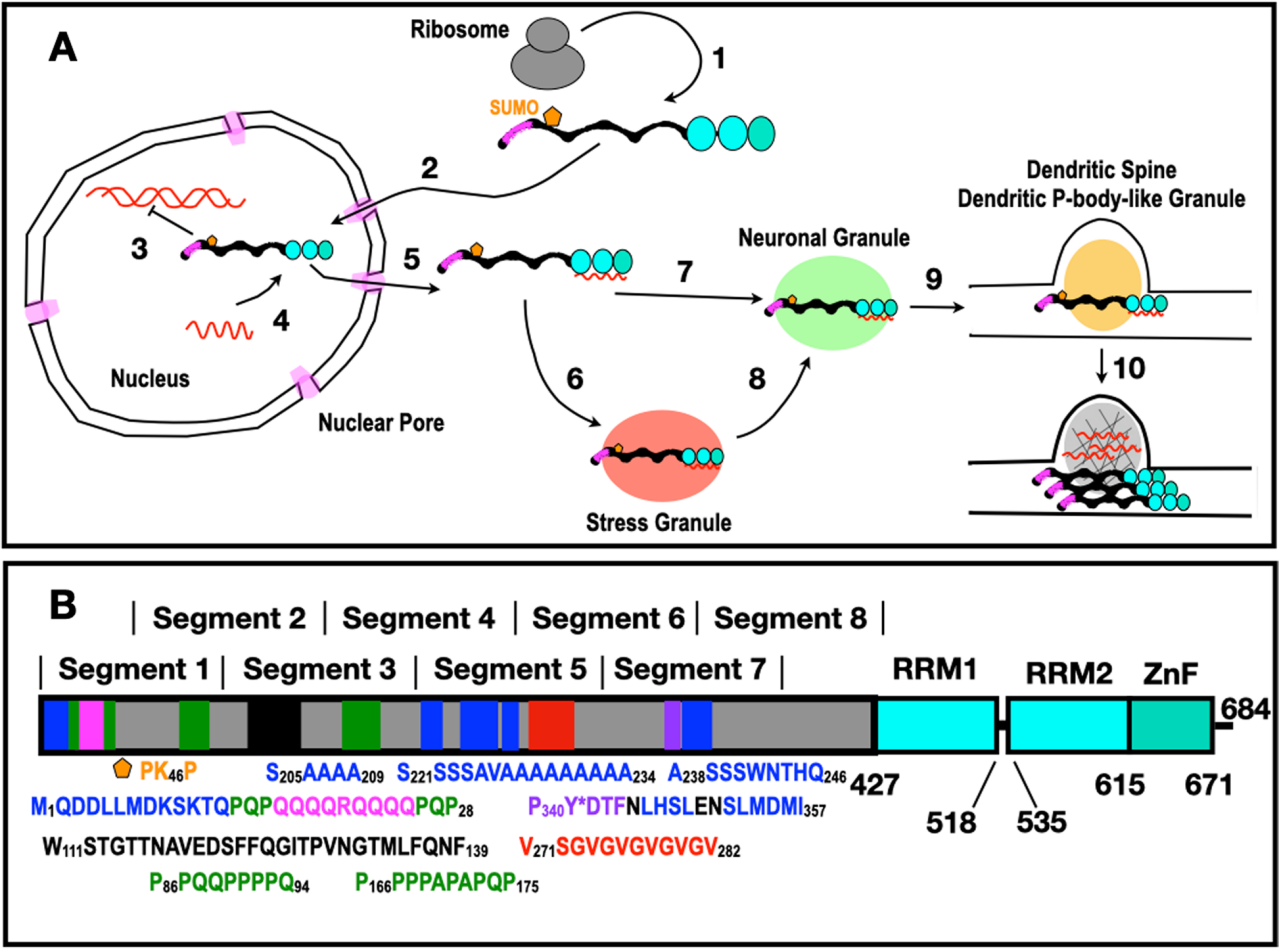
**A** hCPEB3 is present in multiple cellular compartments. Dendritic stimulation leads to temporary, phosphorylation-mediated short-term memory and increased synthesis of the protein CPEB3 (1). Composed of an N-terminal disordered region (black) which includes a Q-rich segment aiding functional aggregation (magenta), hCPEB3 also contains RRM domains (cyan) and a ZZ-Zinc finger domain (turquoise). Upon continued neuro-stimulation, CPEB3 enters the nucleus through the nuclear pore (light magenta), which is a macromolecular condensate (2). Once in the nucleus, CPEB3 indirectly regulates transcription through STAT5B (3) and binds to certain mRNAs (4, red). This binding suppresses translation. After exiting the nucleus through the nuclear pore, (5) CPEB3 + mRNA may associate with a stress granule (6, rose) during moments of adverse conditions. In the absence of stress (7) or its passing (8), CPEB3 + mRNA will combine with another condensate called neuronal granules (light green) for transport to dendritic spines (9), where CPEB3 + mRNA associate with still another class of condensate called a dendritic P-body-like structure (golden) [21]. Further neuronal stimulation (10) causes synapse-specific deSUMOlyation, CPEB3 aggregation, and translational activation of previously repressed mRNA, leading to morphological changes and fortification of the spine, which is proposed to be the basis of long-term memory. This is a simplified model based on that of Kandel and coworkers [22]. **B** CPEB3 domain composition and its N-terminal intrinsically disordered domain (gray) contains key elements with preferred conformers colored blue for α-helix, magenta for polar amyloidogenic, black for hydrophobic amyloidogenic, green for PPII helix, purple for the putative phosphoTyr site, and red for highly disordered segments. The two RRM domain are colored cyan and the C-terminal Zinc Finger is shown in turquoise

Mammalian CPEB3 travels to distinct neuronal regions to carry out multiple functions, where the 426-residue long IDR plays a key role (Fig. 1A). Following its synthesis, CPEB3 is SUMOlyated, which has been reported to block CPEB3 aggregation [14]. Upon neuronal stimulation, CPEB3, which is mostly cytoplasmic, travels to the nucleus. This process is mediated by the karyopherin IPO5 through interactions with the NLS in the first RNA recognition motif (RRM) of hCPEB3 [15]. Inside the nucleus, CPEB3 interacts with STAT5B, which normally activates the transcription of genes such as *EGFR*, triggering signaling cascades thought to promote memory consolidation [16, 17]. CPEB3-STAT5B binding, driven by interactions between the IDR of hCPEB3 and residues 639–700 of STAT5B, downregulates STAT5B-dependent transcription [17]. However, the details of this interaction have not yet been addressed. By contrast, the 3D structure of the first CPEB3 RRM domain has been elucidated and revealed a β-hairpin (W471-G485) proposed to play a key role in RNA recognition [18]. This domain, as well as the second RRM domain and zinc finger (ZnF) motif, were reported to bind specifically to the 3′UTR mRNA of the AMPA receptor subunit GluR2 [19]. Together, CPEB3 and its target mRNA eventually exit the nucleus and can join distinct biomolecular condensates such as stress granules and neuronal granules, which provide physiological transport to dendritic spines, or to dendritic P-body-like granules [20], where CPEB3 stores GluR2 mRNA and downregulates its translation [19].

After synaptic activity in the hippocampus, SUMOylation of CPEB3 decreases [14] and CPEB3 converts, mediated by the IDR, from a translation repressor into a self-sustaining activator, promoting the translation of AMPA receptors [12]. This leads to structural modifications, including a more robust actin network, which fortify the spine and permanently enhance neurotransmission at a given particular synapse [13]. The hypothesis that CPEB3 functional amyloid formation is key for memory persistence in mammals is supported by the impairment in long-term memory and long-term potentiation in the CPEB3 conditional knockout mice [12]. In our species, the causal role of human CPEB3 (hCPEB3) in memory is corroborated by observations that persons carrying a rare CPEB3 allele, which leads to a decreased production of hCPEB3 protein, have episodic memory impairments [23].

The 426-residue long IDR of hCPEB3 plays a key role mediating memory persistence through this prion-like mechanism. It contains an amyloid-forming region spanning residues 1–200 and a condensate-promoting region formed by residues 250–426 which are linked by an alanine rich segment [24]. The full IDR is followed by two folded RRM which bind RNA and finally a ZZ-type ZnF domain (Fig. 1B). Recent sequence and deletion mutational analyses of the IDR have begun to identify subregions key for aggregation, such as the first 30 residues [13]. However, for the hCPEB3 IDR, programs to predict secondary structure tendencies give different outputs, Alpha Fold 2 structural predictions [25] are marked as low to very low confidence (see https://alphafold.ebi.ac.uk/entry/Q8NE35), and, to date, no high-resolution experimental data on the partial structures or motions have been reported. Here, motivated by the key roles of the IDR in CPEB prion-like aggregation required for memory persistence, we characterize the atomic level conformations and dynamics of the complete IDR of hCPEB3 by NMR spectroscopy.

## Results

### hCPEB3’s IDR is chiefly disordered

As a first step to experimentally characterize hCPEB3’s IDR, we probed the complete 426-residue IDR of hCPEB3 by biophysical techniques and homonuclear NMR. Its fluorescence emission spectra, recorded at temperatures ranging from 2 to 70 °C, show emission maximum > 350 nm. This is consistent with its six Trp residues being solvent exposed and not buried in the hydrophobic core of a folded domain (Additional File 1. Fig. S1A) [26]. The far UV CD spectra of the hCPEB3 IDR also shows the hallmarks of a disordered protein, namely a minimum near 200 nm [27]. No spectral features indicative of α-helix and β-sheet; namely, minima at 208, 218, or 222 nm and no maximum at 195 nm, are evident (Additional File 1. Fig. S1B). The 1D ^1^H and 2D ^1^H-^1^H NOESY spectra show ^1^H signals clustered into narrow bands near the values observed for short, unstructured peptides (Additional File 1. Fig. S1C) [28, 29]. The sequence alignment of several representative vertebrate CPEB3 proteins using the T-Coffee program is shown in Additional File 1. Fig. S2. Very similar results were obtained from the Omega Clustal program (not shown). Whereas most IDPs show poor levels of sequence conservation, some stretches rich in hydrophobic residues, such residues M1-T12, W111-F139, and Y341-I357, are highly conserved. By contrast, glutamine rich, alanine rich, and some proline rich segments are present only in mammals. Taking all these data together, the presence of large, stably folded domains in the IDR can be ruled out, but short segments with partly populated secondary structures could still be present.

### Atomic level characterization reveals partially structured elements in hCPEB’s N-terminal “disordered” region

To discover and characterize possible segments with partial secondary structure, we applied multidimensional heteronuclear NMR. As the full length IDR is too long to characterize by this methodology, we have followed the “divide and conquer” approach implemented by Zweckstetter et al. to characterize tau, a similarly sized IDP implicated in Alzheimer’s disease and other tauopathies [30]. As described in the “Methods” section, and shown in Table 1, eight overlapping segments of 100 residues were characterized.

Using our powerful ^13^CO, ^15^ N, ^1^HN-based assignment strategy, over 99% of the main chain ^13^CO, ^13^Cα, ^15^ N, ^1^HN, and the ^13^Cβ resonances were assigned for residues 1–450 of hCPEB3. The chemical shifts of the complete IDR of hCPEB3 are reported in the BMRB (entry number 50256), and the original 2D and 3D spectral data have been deposited in the Mendeley data repository. The assigned 2D ^1^H-^15^ N HSQC and 2D ^13^CO^15^N spectra of segment 4 are shown in Additional File 1. Fig. S3 and Additional File 1. Fig. S4, respectively. The 2D ^1^H-^15^ N HSQC spectra of segments 1, 3, 4, 5, 6, 7, and 8 are shown in Additional File 1. Fig. S5. The similar positions of most ^1^H-^15^ N signals in neighboring segments additionally suggest a sparsity of long range interactions under these conditions. Likewise, the majority of the crosspeaks of the same residues in adjacent segments also overlap or are close together in the 2D ^13^CO^15^N spectra of segments 1, 3, 4, 5, 6, and 8 (Additional File 1. Fig. S6).

Multiple attempts to express and purify hCPEB3 segment 2, which spans residues 51–150, by recombinant methods were unsuccessful. Nevertheless, all the residues within segment 2 are present and have been characterized structurally in the context of segments 1 and 3. To test if there might be some structure in the neighborhood of residues 91–110 located in the middle of segment 2 and the C- and N-termini of segments 1 and 3, respectively, we studied the conformation of a twenty-residue peptide corresponding to this region by NMR spectroscopy. No significant trends towards structure formation were detected (Additional File 1. Fig. S7).

The ^13^Cα and ^13^CO conformational chemical shifts (Δδ) of the hCPEB3 segments 1 and 3–8 are plotted in Additional File 1. Fig. S8. These data show five segments, comprising residues 1–10, 202–210, 222–234, 238–246, and 346–356, with significantly high Δδ^13^Cα and Δδ^13^CO values. Such values are characteristic of partially populated α-helices and are examined in detail in the following paragraphs.

### The first residues of hCPEB3 adopt a partly populated α-helix which precedes the Q-rich stretch

**Fig. 2:**
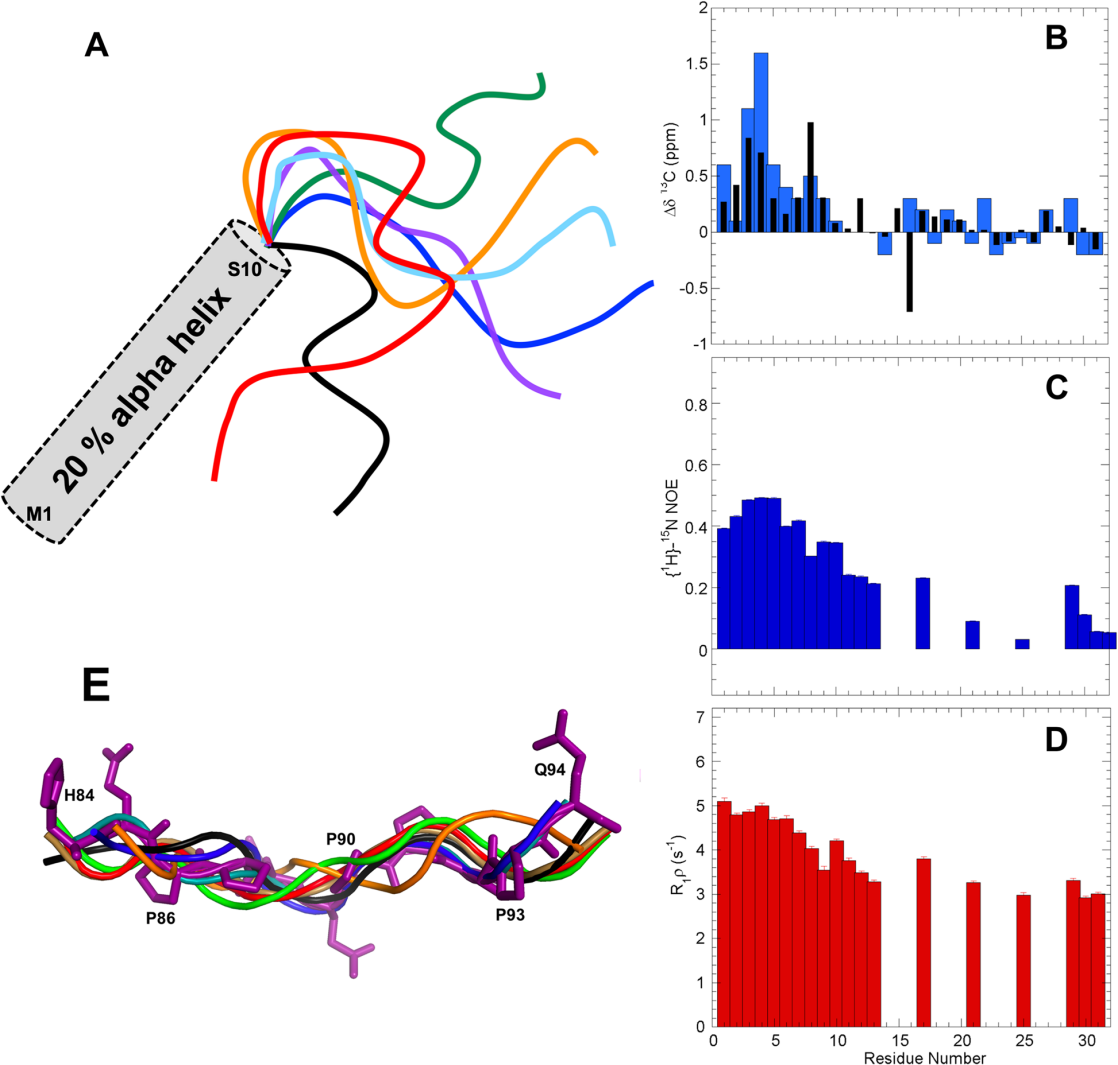
The N-terminal 25 residues of hCPEB3 adopt a hydrophobic α-helix followed by a disordered polyQ segment flanked by PQP mini-breaker motifs. The N-terminus of hCPEB3 contains an α-helix-forming and a disordered amyloidogeneic Q_4_RQ_4_ segments, which are separated by PQP mini breaker motifs. **A** Schematic representation as a gray cylinder of partial (20%) α-helix formation by the first ten residues of hCPEB3. The disordered conformational ensemble of residues 11–32 is represented curved lines colored purple, blue, cyan, green, orange, red, and black. **B** ^13^Cα (blue) and ^13^CO (black) conformational chemical shifts indicate a 20% population of helix at 25 °C. Uncertainties in the conformational chemical shifts (Δδ) are 0.02 and 0.10 ppm for ^13^CO and ^13^Cα, respectively. **C** {^1^H}-^15^ N NOE and **D** R_1_ρ relaxation measurements indicate that this helical conformation is less mobile than the polyQ segment at ns/ps and µs/ms timescales, respectively at 25 °C. Error bars are shown in **C** and **D** but are small as the estimated uncertainties are < 0.01 for the hNOE and < 0.1 s^−1^ for R_1_ρ. Missing values in **C** and **D** are due to overlap of ^1^H^15^N peaks or a lack of ^1^H^15^N signals in the case of proline residues (see Additional File 1. Fig. S9 for additional values from ^13^C-detected relaxation experiments). **E** Eight representative backbone conformers, colored purple, blue, teal, green, amber, orange, red, and black, of the proline rich segment, H84-Q94, featuring a PPII helix that spans residues P86-Q94. All heavy atoms are shown for the purple conformer

The conformational chemical shifts point to the formation of partly populated (20%) α-helix in the first ten residues of the protein (Fig. 2A, B). Increased conformational chemical shifts are observed at 5 °C, reflecting a higher amount of helical structure upon cooling. Standard ^1^H-detected ^15^ N relaxation measurements detect that these residues are the most rigid part of segment 1 (Fig. 2C, D). These results are corroborated by ^13^C-detected ^15^ N relaxation experiments (Additional File 1 Fig. S9).

The α-helix detected for these first residues extends N-terminally into the His/Tev tag. To rule out a possible structure-promoting effect on segment 1, we tried to remove it by proteolytic cleavage with the TEV protease. Multiple attempts failed, which suggests that the helix spanning the last residues of His/Tev tag and the first residues of the hCPEB3 IDR is present and impedes the proteolytic cleavage. Therefore, we characterized a dodecamer peptide whose sequence corresponds to the first twelve residues, M_1_QDDLLMDKSKT_12_, of the hCPEB3 IDR. The observation of a series of weak ^1^HN_i_—^1^HN_i+1_ nuclear Overhauser enhancement (NOE) crosspeaks reveals that this peptide has a slight tendency to form α-helix in aqueous buffer (Additional File 1. Fig. S10A). Fluorinated alcohols like trifluoroethanol (TFE) and hexafluoroisopropanol (HFIP) are known to increase the population of helical conformations in peptides which have an α-helix forming tendency, but not in peptides which prefer to adopt β-strands or random coil [31]. In the presence of 20% HFIP, the population of α-helix in this peptide increases strongly, based on the observation of stronger and more numerous NOE crosspeaks as well as ^1^Hα and ^13^Cα conformational chemical shifts (Additional File 1. Fig. S10B). These findings evince that the first 12 residues of hCPEB3 do tend to adopt an α-helix.

Interestingly enough, the polyQ segment, Q_16_QQQRQQQQ_24_, does not form an α-helix or a β-strand and appears to be thoroughly disordered and flexible (Fig. 2A, B). A construct spanning residues 1–200 of hCPEB3, which contains the Q_4_RQ_4_ motif, plays a role in hCPEB3 amyloid formation as indirectly evidenced by the anti-amyloid action of the polyglutamine-binding peptide 1 (QBP1) [24]. This polyQ segment is preceded and followed by Pro-Gln-Pro residue triplets (P_13_QP_15_ and P_25_QP_27_). Considering the inhibitory effect of proline residues previously observed for polyQ amyloid formation in Huntingtin by Wetzel and co-workers [32], it is likely that these PQP mini-motifs check amyloidogenesis by the polyQ segment. The first 100 residues also contain a predicted SUMOylation site [24] at Lys 47 and ends with a proline-rich segment P_86_PQQPPPPQEPAAPG_100_, which is associated with solubility. Whereas recently reported NMR criteria [33] allow us to rule out that this stretch folds into a stable polyproline II (PPII) helical bundle, the steric limitations of polyproline segments mean that residues 86–93 adopt an isolated, partly populated PPII helix (Fig. 2E). In fact, the consecutive proline residues show a distinct pattern of conformational chemical shifts; namely + 0.6 ppm, − 1.0, and − 0.3 for ^13^Cα, ^13^Cβ, and ^13^CO, respectively (Table 2, Additional File 1 Fig. S11). Not observed in isolated proline residues, we advance that they are hallmarks of a PPII helical conformation.

**Table 2:** Conformational chemical shifts for α-helices, β-strands, and PPII helices

	α-Helix	β-Strand	Gly-rich PPII helical bundle	Isolated Pro-rich PPII helix
δΔ ^13^Cα	3.1^a^/2.9^b^	− 1.5^a^/ − 1.8 ^b^	− 0.6^d^	+ 0.6^e^
δΔ ^13^Cβ	− 0.4^a^/ − 0.8^b^	+ 2.2^a^/ + 2.7^b^	+ 0.3^d^	− 1.0^e^
δΔ ^13^CO	2.2^c^/2.2^b^	− 2.2^c^/ − 2.1^b^	− 0.2^d^	− 0.3^e^

^a^From Spera and Bax (1991) [34]

^b^From Wang and Jardetzky (2002) [35] for alanine

^c^Calculated from Wishart and Skyes (1994) [36]

^d^From Treviño et al. (2018) [33]

^e^This study

### Residues 101–200 of hCPEB3 contains a rigid nonpolar segment and a PPII helix

Regarding residues 101–200, no strong trends to adopt α-helical or β-structures are detected. Nevertheless, the stretch composed of residues, W_111_STGTTNAVEDSFFQGITPVNGTMLFQNF_139_ which contain numerous aliphatic and aromatic residues, shows relatively high rigidity, both on fast ns/ps as well as slower µs/ms timescales (Additional File 1. Fig. S12). This finding is interesting considering that this relatively hydrophobic segment also appears to be essential for hCPEB3 amyloid formation in vitro [24], and very recently, it has been reported to form amyloid in mouse CPEB3 [37]. In addition, the stretch of residues 161–190 Q_161_HHQQPPPPA_170_PAPQPAQPAQ_180_PPQAQPPQQR_190_ has a very high Q/P content, and Pro and Gln are the residues with the highest intrinsic tendencies to adopt PPII helices [38]. The consecutive proline residues, P_166_PPPAPAPQP_175_, also display the characteristic PPII pattern of conformational chemical shifts (Additional File 1. Fig. S11 ABC) seen for residues 86–93. Although more weakly than long stretches of pure polyproline [39, 40], a synthetic peptide corresponding to residues P166-P175 of hCPEB3 binds to human Profilin 1, a known mediator of interactions with actin (Additional File 1. Fig. S11 D).

### Residues 201–300 contain three α-helical segments and a disordered (VG)_5_ segment

**Fig. 3:**
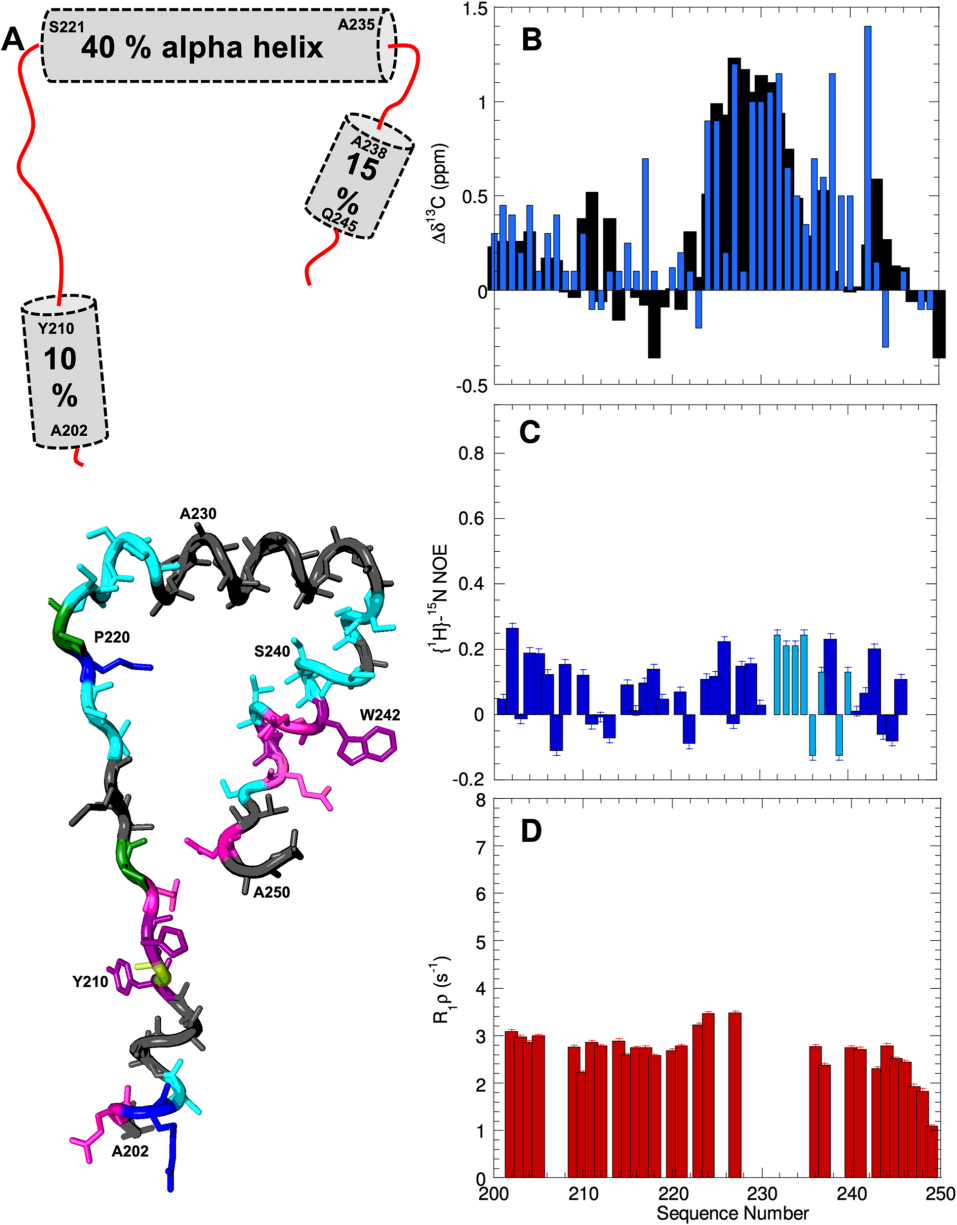
Residues 201–250 adopt three partial populated α-helices. **A** (*Top*) Schematic representation as gray cylinders of the three partially populated helices present in residues 200–250. (*Bottom*) One conformer with all three α-helices is shown; residues are colored: cationic residues (R and K) = blue, aromatics (F, Y, W and H) = purple, anionic (E and D) = red, aliphatic (A, I, L, M) = dark gray, amyloidogenic (N and Q) = magenta, hydroxyl bearing (S and T) = cyan, and proline = green. **B** ^13^CO (black) and ^13^Cα (blue) conformational chemical shifts (Δδ) of residues 201–250 at 25 °C. Note that the second α-helix which contains nine consecutive Ala residues has a relatively high helical population. Uncertainties in the conformational chemical shifts (Δδ) are 0.02 and 0.10 ppm for ^13^CO and ^13^Cα, respectively. **C** {^1^H}-^15^ N NOE ratios. Values shown in dark blue are of individual ^1^H^15^N resonances; those in light blue correspond to overlapped peaks. **D** R_1ρ_ values reveal the ps/ns and µs/ms time scales. Significantly higher {^1^H}-^15^ N NOE ratios and R_1ρ_ values are observed for these residues at 5 °C (Additional File 1. Fig. S10)

Significant ^13^Cα and ^13^CO chemical shift deviations with respect to values predicted for a statistical coil, for three residue segments spanning A_202_QRSAAAY_**21**0_ GHQPIMTSKP_220-_S_**221**_SSSAVAAAA_230_AAAAA) SSASS_240_SWNTHQSVHAA_250_ (Fig. 3A). These results indicate that the three segments of underlined residues adopt partially populated α-helices. Based on the magnitude of the conformational chemical shifts, the helical populations are different, being about 30% for the A_202_-Y_210_ α-helix, 80% for the S_222_-A_234_ α-helix and 20% for the A_238_-Q_246_ α-helix at 5 °C; whereas these populations decrease at 25 °C to approximately 10%, 40%, and 15%, respectively, they are still significant (Fig. 3B). The presence of the first and second helices are confirmed by ^1^HN-^1^Hα coupling constants (Additional File 1 Fig. S13). Moreover, analysis with TALOS + , which predicts secondary structure taking into account ^13^Cβ, ^15^ N and ^1^Hα chemical shifts in addition to ^13^Cα and ^13^CO, confirms the presence of these three helical segments and structural calculations with CYANA suggest that the three helices do not tend to adopt a preferred alignment relative to each other (data not shown). The helices are not especially rigid on fast ps-ns time scales (Fig. 3C) or the slower µs-ms time domain (Fig. 3D) at 25 °C but do show a heightened stiffness at 5 °C (Additional File 1. Fig. S12). Helical wheel projections (Additional File 1. Fig. S14) suggest that different interactions contribute stability to these α-helices. Gly 211 and His 212 are positioned to stabilize the A_202_-Y_210_ α-helix by a C-capping motif [41]. Whereas Ala has a very high intrinsic helix forming propensity, the propensity of Ser is low [42]. In this segment, however, the Ser residues are positioned at the N-terminus of the α-helices, where adding negative charge via phosphorylation would increase the helical population, considering the well-known stabilizing effects of charge/macrodipole interactions and N-capping H-bonds [43]. This proposal is supported by NMR spectroscopic characterization of a peptide EAVAAAAAAAKK, with a phosphomimetic N-terminal Glu residue, which reveals a modest increase in helicity as the pH is raised from three, where the Glu is mostly neutral to five, where the Glu is chiefly anionic (Additional File 1. Fig. S15). Although this sequence’s insolubility thwarts attempts to more directly test the impact of phosphorylation, we note that these Ser residues are placed at the positions where phosphorylation is expected to increase α-helix stability the most [44]. Moreover, at neutral pH, where phosphoserine carries two negative charges, the stabilization is substantially greater than at pH 4, where it carries one [44]. The last α-helix, A_238_-Q_246_, is less populated, but its stability might increase if W242 were to engage in long-range interactions, such as with the hydrophobic or cationic residues of the first α-helix, i.e. MQDDLLMDKSKT. To test this possibility, we studied two polypeptides containing the M_1_-T_12_ and A_238_-Q_246_ helical segments with and without an N-terminal Dansyl group, connected by a flexible (Gly)_4_ linker. The results of FRET and 2D NMR spectroscopy evince that this polypeptide adopts a conformational ensemble significantly more compact than a statistical coil but that the α-helix contents are not significantly altered (Additional File 1. Fig. S16).

One of the most striking features in hCPEB3’s sequence is a short dipeptide protein motif (Val-Gly)_5_ spanning residues 271–281, which is reminiscent of longer (Ala-Gly)_N_ and (Pro-Gly)_N_ and (Arg-Gly)_N_ dipeptide repeat proteins encoded by mutant C9orf72 which have been implicated in ALS [45, 46]. Our in silico analysis identified this segment as having a high potential to form amyloid [24]. In the context of hCPEB3, however, this segment is among the most disordered and flexible of all the zones of the IDR (Additional File 1. Fig. S8, S12). Just beyond the (VG)_5_ segment, there is a stretch of 15 residues, S_284_PLNPISPLKKPFSS_298_, whose NMR parameters indicate disorder and flexibility (Additional File 1. Fig. S8, S12). Nevertheless, this stretch contains four Ser residues reported to phosphorylated by protein kinase A (PKA) or calcium/calmodulin-dependent protein kinase II [47] (Table 1) and therefore might be important for the transition between short- and long-term memory. Residues P_303_-PKFPRAAP_311_ are proline rich. Predictions suggest that Arg 308 can be methylated (Table 1). This modification, whose impact has not been probed here, was reported to fortify cation–π interactions, reduce interactions with RNA, and destabilize condensates in other proteins [48].

### The residues forming the Nuclear Export Signal (NES) show a marked tendency to adopt α-helical structures

**Fig. 4:**
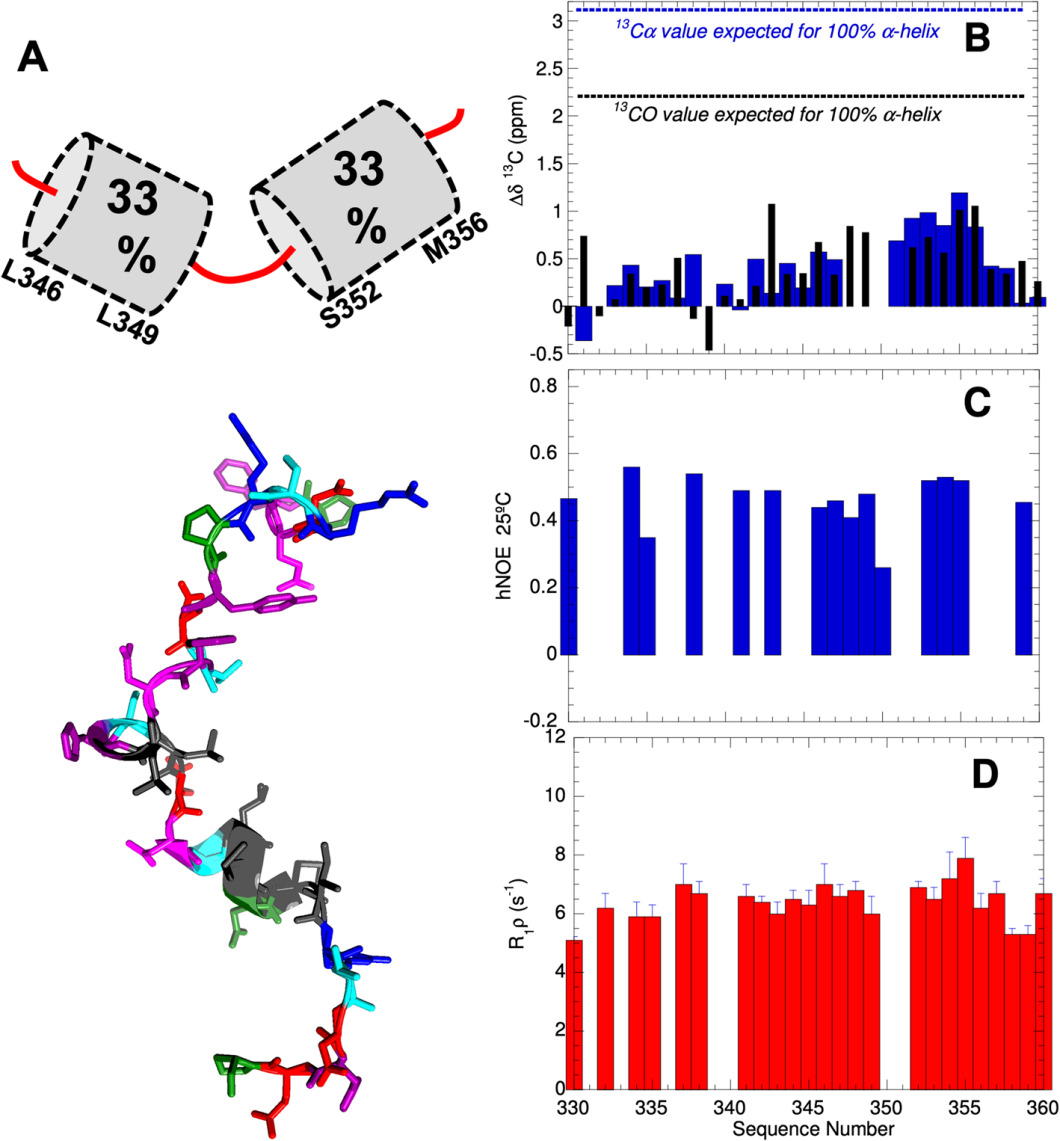
Conformation of the NES and nearby putative phosphoTyr site. **A** (*Top*) Residues L346-L349 and S352-M356 adopt two short, partially populated α-helices. (*Bottom*). Representative conformer is shown with cationic residues (R and K) colored blue, aromatics (F, Y, and H) = purple, anionic (E and D) = red, aliphatic (I, L, M) = dark gray, amyloidogenic (N and Q) = magenta, hydroxyl bearing (S and T) = cyan, and proline = green. Spiral ribbons mark the helical segments spanning residues 346–349 and 352–356. **B** Conformational chemical shifts of ^13^Cα (blue bars) and ^13^CO (black narrow bars) afford detection of helical conformations. Uncertainties in the conformational chemical shifts (Δδ) are 0.02 and 0.10 ppm for ^13^CO and ^13^Cα, respectively. **C** {^1^H}-^15^ N NOE ratios of 0.85 and − 0.20 are indicative of high rigidity and flexibility, respectively, on ps-ns time scales. **D** Higher R_1ρ_ rates are diagnostic of rigidity on µs-ms time scales

Significant conformational chemical shifts were also observed for residues L349-L353 which form the NES (Fig. 4) indicating the presence of helical structure. Using the ^13^CO, ^15^ N, ^1^HN, ^13^Cα, and ^13^Cβ chemical shift data as input, a family of conformers was calculated using the programs TALOS + and CYANA for residues P333-P363. This 31-residue segment is rich in aromatic (five) and aliphatic (six) residues, which is unusual for a disordered polypeptide. The resulting structures reveal that residues L346-L349 adopt one turn of α-helix and residues S352-M356 form a short α-helix (Fig. 4A). It is notable that this conformer positions five nonpolar residues: L346, L349, L353, M354, and I357 on the same face of the α-helices. Y341, the putative phosphorylation site, is in an extended portion of the backbone and would be accessible for this post-translational modification (PTM). Whereas the conformational ensemble will contain many other structures, based on conformational chemical shifts as illustrated by the Δδ^13^Cα and Δδ^13^CO values shown in Fig. 4B, the α-helical population is about one third. The presence of rigid conformers is corroborated by relatively high {^1^H}-^15^ N NOE ratios (Fig. 4C) and elevated transverse relaxation ratios (Fig. 4D).

Beyond the NES α-helix, no segments with preferred secondary structure are detected. The last residues of the segment 8 construct S_426_-RKVFVGGLPPDIDEDEITASFRRF_450_ belong to the RRM1 domain. According to the 3D structure [18], residues K428–G432 adopt a β-strand and residues E440–R449 form an α-helix in the context of the complete RRM1 domain. Here, these segments appear to be largely disordered. After a proline-rich zone ending around residue 380, the next fifty residues have a higher content of nonpolar residues and tend to be more rigid (Additional File 1. Fig S12). Residues 400–412 SHGDQALSSGLSS contain five Ser residues reported to be phosphorylated [47] (Table 1).

## Discussion

Like its homologs in *Aplysia* and *Drosophila*, hCPEB3 resembles the diverse superfamily of RNA-binding proteins that contain RRM and/or ZnF domains as well as intrinsically disordered prion-like regions, such as fused in sarcoma (FUS) or transactive response DNA-binding protein of 43 kDa (TDP-43). FUS and TDP-43 are essential proteins, but their anomalous aggregation has been implicated in amyotrophic lateral sclerosis (ALS) and frontotemporal dementia (FTD). Thus, comparing the CPEB3 and TDP-43 IDR conformational tendencies and dynamics may reveal why the latter can become pathological. The biophysical analysis of the full length hCPEB3 IDR shows that it lacks stable secondary structure. Disordered regions tend to change their sequence much more rapidly over the course of evolution due to a lack of structural constraints [49]. The strong conservation in vertebrate CPEB3 of the N-terminal α-helix and the 350’s (NES) α-helix and the nearby putative phosphoTyr site suggests physiological importance (Additional File 1. Fig. S2). The latter’s hypothetical binding to the SH2 domain of STAT5B might occlude the NES, leading to nuclear retention. Additional segments, like the hydrophobic stretch key for amyloid formation [24] and a cluster of three Trp residues (W242, W252 and W259), are also conserved from mammals to fish. In contrast, the N-terminal Gln-rich segment, the Pro-rich “breaker” regions and the Ala-rich helices are well conserved in mammals but not across all vertebrates. In some lower vertebrates, there is an alternative Q-rich region positioned after the 100’s hydrophobic segment. These elements’ rapid evolution could be related to the development of the mammalian brain. By contrast, the ability to move the poly-Q segment or substitute it for a hydrophobic amyloidogenic segment highlights the cassette or modular nature of PLDs, which was previously established for the *Drosophila* CPEB homolog [5].

### PPII helices may regulate amyloid formation

Whereas hCPEB3 and its homologs Orb2 and *Ap*CPEB have α-helix destabilizing residues between the N-terminal α-helix and the amyloidogenic Q-rich segment, such as proline in hCPEB3 and Orb2 or serine and valine in the case of *Ap*CPEB, no such α-helix busters are present in the analogous segments of huntingtin, the androgen receptor, or TDP-43 (Table 3). This allows the poly-Q segment to interact with the α-helix through the formation of sidechain to backbone hydrogen bonds in the androgen receptor [50] and to form part of the amyloid structure as proposed for TDP-43 [51] and found in ALS/FTLD patient brains [52]. The presence of α-helix breakers between the α-helix and the poly-Q(/N) segments in functional amyloids and their absence in pathological amyloids may be a fundamental difference behind their radically different toxicities.

**Table 3:**
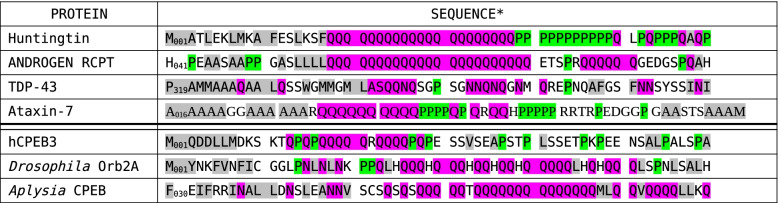
Hydrophobic helices and Pro rich stretches modulate amyloid formation by Q-, Q/N-rich segments

^*^In these sequences, nonpolar residues are shaded gray, Gln and Asn residues are shaded magenta, and prolines are shaded green

PPII helices, well known in collagen and certain glycine-rich proteins [53], also play key roles in mediating protein–protein interactions in biomolecular condensates [54]. Whereas conformation chemical shifts have proven to be extremely useful tools to identify α-helices and β-strands in folded and disordered proteins, conformational chemical shifts for PPII helices were less known. The first 450 residues of hCPEB3 include no less than 66 proline residues (15% of the total). We hypothesize that segments such as G_165_PPPPAPAPQP_175_ may bind profilin, which has specific domains to interact with proline-rich polypeptides [39] and to mediate interactions with actin, whose levels rise at the synapse following CPEB3 aggregation [13]. On the basis of the thorough set of chemical shifts obtained here, including Pro ^15^N assignments which are rare in the literature, we propose a pattern of conformational chemical shifts that define PPII helices (Table 2). With standards for glycine-rich PPII helical bundles [33], these values should aid the detection of PPII helices in biomolecular condensates and the elucidation of their roles in physiology and pathology.

### Towards an atomic-level description of the first steps in hCPEB3 IDR self-association

**Fig. 5:**
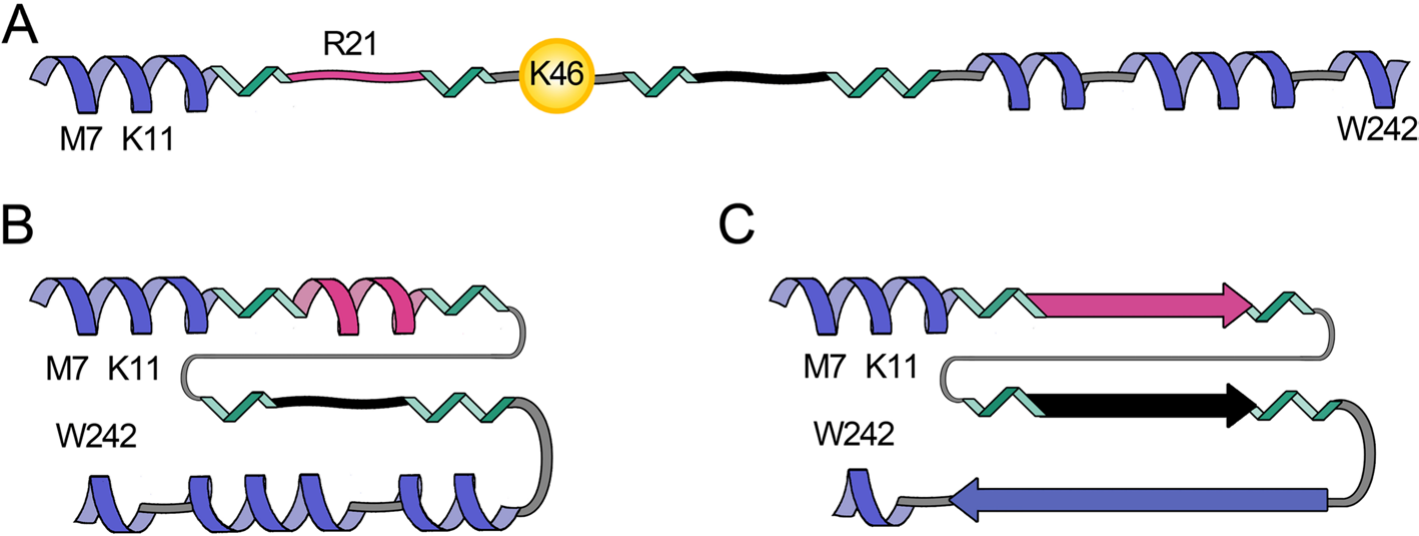
Working hypothesis for hCPEB3 structural changes during memory consolidation. **A** The first 250 residues of hCPEB3 contain 4 α-helices (blue spirals); the first and third α-helices are relatively stable. Proline-rich segments (green) and SUMOylation as predicted to occur at K46 by in silico methods [24] prevent premature association and amyloid formation by the Q_4_RQ_4_ segment (magenta) and the hydrophobic motif (black squiggle). **B** Following deSUMOlyation and putatively phosphorylation, association between the fourth and first helices could occur, strengthened by hydrophobic and cation-π interactions. The Q_4_RQ_4_ segment may adopt an α-helix and associate with the Ala rich α-helices to form a coiled-coil. The structural transformations may well enhance intermolecular contacts within the dendritic P-body like granule, leading to gelification and, eventually, amyloid formation. **C** The final amyloid could be composed of the Q_4_RQ_4_ segment and hydrophobic tract and possibly the Ala-rich segments. The final configuration of the polyPro segments (green) may promote profilin binding and the initiation of a more robust actin filament network

Based on the NMR results, we suggest a speculative working hypothesis for hCPEB3 conformational changes (Fig. 5). Initially, the first 200 residues of hCPEB3, which are necessary and sufficient for aggregation and amyloid formation in vitro [12, 24], are mostly disordered except for the relatively stable α-helices formed by residues 222–234 and modestly populated α-helices at the N-terminus and spanning residues 202–212 and 237–245 (Fig. 5A). The Q_4_RQ_4_ motif and hydrophobic segment F_123_FQGIT-PVNGT-MLFQNF_139_ are initially disordered and premature amyloidogenesis is discouraged by SUMOlyation [14] and proline breaker motifs. Association among the α-helices into more compact ensembles, as suggested by FRET results (Additional File 1. Fig. S16), would become possible and might be strengthened by hydrophobic interactions between Met 7 and Trp 242 or cation-π interactions between Lys 11 and Trp 242 (Fig. 5B), analogous to the long range contacts seen in a TDP-43 amyloid [55]. The increased production of hCPEB3 upon neuronal stimulation [12] as well as the proximity of the serine and alanine rich α-helix (residues 222–234) may promote α-helix formation by the QQQQRQQQQ motif. These could then associate to form a coiled-coil, as has been demonstrated in model polypeptides [56–58]. Moreover, completely helical polyalanine peptides, some of which are linked to polyalanine expansion diseases, were reported to promote coiled-coil mediated aggregation, and no conversion into β-sheet structures was observed [57, 59]. Although future studies are necessary to provide additional evidence and test plausible hybrid aggregation mechanisms with several elements of structure acting in parallel, this model is supported by analogous results on *Ap*CPEB [4, 60] and polyalanine expansions [59], which led to the proposal of similar mechanisms.

These events would reinforce intermolecular contacts within the dendritic P-body like granule [20] leading to gelification and, eventually, amyloid formation (Fig. 5C). The final amyloid structure could be comprised of the Q_4_RQ_4_ motif and the hydrophobic segment as evidenced by hCPEB3 fibril formation kinetics [24]. A construct spanning residues 1–200 of hCPEB3, which contains the Q_4_RQ_4_ motif, plays a role in hCPEB3 amyloid formation as indirectly inferred by the anti-amyloid action of QBP1 [24]. For the hydrophobic segment, its role in mouse CPEB3 amyloidogenesis has recently been corroborated [37]. In addition, the poly-A segment of helices 202–212 and 222–234 may also transform into amyloid (Fig. 5C). Such α-helix to amyloid conformational transformations have been previously described in alanine-rich polypeptides as diverse as a fish antifreeze protein [61] and a synthetic (Ala)_10_-(His)_6_ hexadecapeptide [62] and could form pathological amyloids in several diseases involving polyA expansions [63].

Residues 217–284 of CPEB3 have been reported to be key for interactions with actin [13]. Considering this, the hypothetical associations among the α-helices formed by residues M_1_–T_12_ and residues A_202_–Q_246_ could dispose the PPII helices formed by residues P_86_–Q_94_ and P_166_–P_175_ (Additional File 1. Fig. S10) to bind profilin. This protein contains a second binding site specific for actin and promotes actin filament network formation [64]. Such actin networks are known to become more robust as a dendritic bud strengthens during memory consolidation [65].

## Conclusions

In summary, by detecting several partly populated α-helices, PPII-helices, and hydrophobic segments in the hCPEB3 IDR, these NMR spectroscopic results provide clues for comprehending the first structural transitions involved in protein/protein and protein/RNA interactions which may be key for memory consolidation as well as motifs that discriminate functional versus pathological amyloids.

## Methods

### Materials

^15^NH_4_Cl and ^13^C-glucose were purchased Tracertec (Madrid, Spain); D_2_O was a product of Euroisotop. Deuterated acetic acid was from Sigma/Aldrich, and 4,4-dimethyl-4-silapentane-1-sulfonic acid (DSS) as the internal chemical shift reference, was from Stolher Isotopes Chemical Company.

A twelve residue peptide, called hCPEBpep1, whose sequence corresponds to the protein’s first 12 residues (M_1_QDDLLMDKSKT_12_) and a twenty residue peptide, called hCPEBpep2 with the sequence. P_91_PPQEPAAPGASLSPSFGST_110_ in hCPEB3 were purchased from Genscript. hCPEBpep2’s sequence overlaps with the C-terminus of Segment 1 and the N-terminus of Segment 3. A proline-rich peptide acGPPPPAPAPQPam, with acetylated and amidated termini and whose sequence covers residues 166–175 in hCPEB3, two polypeptides with the sequence MQDDLLMDKSKTGGGGASSSWNTHQ (with and without an N-terminal Dansyl group) were also obtained from Genscript. Finally, another peptide, acEAVAAAAAAAKKam, which corresponds to residues 224–234 but with S224 substituted by E as a phosphomimetic residue and A234 and A235 substituted by K for solubility, was obtained from the Proteome Service of the *Centro Nacional de Biotecnologia*, CSIC. All the peptides were over 95% pure, as assessed by HPLC, and their identities were confirmed by mass spectrometry and NMR spectroscopy. Recombinant Human Profilin 1, produced recombinantly in *E. coli*, was obtained from Abcam (reference number ab87760). It is over 95% pure as judged by SDS PAGE.

### Sample production

Coding mRNAs of hCPEB3 vary in length due to an embedded human delta virus-like ribozyme which slowly splices out introns, leading to the generation of multiple isoforms [66]. It is also noteworthy that Orb2A’s pre-mRNA contains an intron with multiple stop codons which is only spliced out when certain “memorable” stimuli are experienced [9]. Here, the hCPEB3 isoform 2, Uniprot Q8NE35-2/Genebank CAI14105.1, is studied.

### Plasmid construction, protein expression, and purification

The hCPEB3 IDR, which corresponds to the first 426 residues of the protein whose sequence is shown in Table 1, was expressed at eight highly overlapping one-hundred residue segments. To control for end effects and to check the reproducibility, each segment overlaps by 50 residues with the preceding and successive segments.

Each segment was cloned into the pET-28a( +) plasmid by PCR using the full length human CPEB3-2 in pLL3.7 plasmid as the template kindly provided by Dr. Yi-Shuian Huang [67]. The DNA amplified fragments were digested with XhoI and NheI. Expression of the resulting clones led to fusion proteins containing a His_6_ tag and a TEV NIα protease cleavage site. Thus, each segment studied had the sequence MGSSHHHHHHSSGLVPRGSHMASENLYFQ, at its N-terminus.

All overlapping segments were expressed in the *E. coli* BL21 Star (DE3) strain using the T7 expression system (Novagene). ^13^C/^15^N isotopic labeling of each segment was done by using a previously published protocol [68]. Briefly, cells were grown in 1 L of LB at 37 °C by shaking at 280 rpm upon reaching optical cell densities at 595 nm (OD_595_) ~ 0.6–0.7. Cells were pelleted by a 30 min centrifugation at 5000 × *g* and washed using a M9 salt solution (15.0 g/L KH_2_PO_4_, 34.0 g/L Na_2_HPO_4_ and 2.5 g/L NaCl for 1 L of 5 × M9 salts) excluding nitrogen and carbon sources. Cell pellets were resuspended in 250 mL of isotopically labeled minimal media M9 salt solution supplemented with ^13^C D-glucose 4.0 g/L and ^15^NH_4_Cl 1.0 g/L (Cambridge Isotope Laboratories, Inc.) and then incubated to allow the recovery of growth and clearance of unlabeled metabolites. Protein expression was induced after 1 h by addition of IPTG to a concentration of 1 mM. After a 4.5 h incubation period, the cells were harvested.

Cell pellets were lysed with buffer with the following composition: 50 mM NaH_2_PO_4_/Na_2_HPO_4_, 500 mM NaCl, 50 mM imidazole, 6 M guanidinium chloride (GdmCl), pH 7.4, and then sonicated. Each recombinant segment was purified by Ni^++^-affinity chromatography using HisTrap HP purification columns with a FPLC system (ÄKTA Purifier, GE Healthcare) with elution buffer consisting of 50 mM NaH_2_PO_4_/Na_2_HPO_4_, 500 mM NaCl, 500 mM imidazole, 6 M GdmCl, and pH 7.4.

If necessary, the pure segments were then incubated with TEV NIα protease O/N at 4 °C [69]. The cleaved protein was then subjected to dialysis and recovered. Protein samples were stored at − 80 °C until use. Eventually, they were desalted to 1 mM DAc, pH 4.0 by gel filtration chromatography using a PD-10 column (GE Healthcare), and concentrated to a final protein concentration of 1.0–1.5 mM in 250 µL using a Vivaspin microfiltration device and placed in a 5-mm Shigemi reduced volume NMR tube for measurements. These low pH, low ionic strength conditions have been reported [70] and corroborated [71] to maximize solubility by increasing charge-charge repulsion between protein molecules. Intrinsically disordered proteins with large net positive [72] and negative charges [73] have been reported to show highly mobile main chains, either alone or in complex. This strongly suggests that the different net charge on hCPEB3 at pH 4 versus pH 7 is unlikely to significantly impact the local chain dynamics. α-helix stability depends on pH due to charge–charge interactions, charge–helix macrodipole interactions, and intrinsic helix propensities [43]. A consideration of these factors suggests that the effect of increasing the pH from 4 to 7 would be mildly stabilizing for the first α-helix and insignificant for the four remaining α-helices detected in the hCPEB3 PLD.

Non-labeled full length hCPEB3-IDR expression and purification was carried out essentially as described in [24]. Briefly, cells were grown in 1 L of LB medium at 37 °C until reaching an OD_595_ = 0.6–0.7, and protein expression was induced for 4 h by adding IPTG at 1 mM final concentration. Cells were harvested and following sonication, then lysed with buffer 50 mM Na_2_HPO_4_, 500 mM NaCl, 50 mM imidazole, 6 M GdmCl, and pH 7.4. After centrifugation at 18,000 rpm for 45 min, supernatants were purified with Ni^++^ affinity chromatography, and elution was performed in buffer containing 50 mM NaPO_4_, 500 mM NaCl, 500 mM imidazole, 3 M GdmCl, and pH 7.4. Purified CPEB3-IDD was diluted to PBS, 1 M GdmCl pH 7.4, dialyzed against PBS pH 7.4 at 4 °C, and finally concentrated by using Amicon Ultra-15 centrifugal filters with a 10-kDa cutoff.

### Sequence alignment

The conservation of vertebrate CPEB3 protein sequences was assessed using the programs T-coffee [74] and Clustal Omega [75] using the default settings. The sequences chosen as representative are human isoform 1 NP_001171608.1, human isoform 2 CAI14105.1, mouse NP_001277755.1, chicken XP_105144323.1, turtle (*Chrysemys picta belli*) XP_005301348.1, frog NP.001015925.1 (*Xenopus tropicalis*), and fish (*Danio rerio*) XP_009305819.1.

### Fluorescence and circular dichroism spectroscopies

Fluorescence spectra on the full length, unlabeled hCPEB3-IDR at ca. 5 μM in 1.0 mM deuterated acetic acid at pH 4.0 were recorded on a Horiba FluorMax 4 instrument equipped with a Peltier temperature control device using a 0.2 s·nm^−1^ scan speed and three nm excitation and emission slit widths. The excitation wavelength was 280 nm, and the emission was scanned over 300–400 nm. A series of spectra were recorded at 2, 10, 20, 30, 40, 50, 60, and 70 °C.

To test for binding of a segment of hCPEB3 rich in proline residues to Profilin, fluorescence spectra were recorded at 20 °C on 10 μM human Profilin 1 using 2-nm excitation and emission slits, a 2-nm·s^−1^ scan speed, and an excitation wavelength of 295 nm, and emission was recorded from 300 to 400 nm in the absence and presence 4.5 mM of the peptide acGPPPPAPAPQPnm, which corresponds to residues P166-Q175 of hCPEB3, with an N-terminal acetyl and glycine residue and a C-terminal amide group added to avoid end charges. This assay is based on blue shift and enhanced emission of Trp3 and Trp31 of human Profilin 1 as the environment surrounding their indole moieties becomes less solvent exposed upon polyproline ligand binding [39].

Fluorescence spectra on Dansyl-MQDDLLMDKSKTGGGGASSSWNTHQ and the control polypeptide without Dansy, MQDDLLMDKSKTGGGGASSSWNTHQ, were recorded using an excitation wavelength of 295 nm, which is selective for the donor Trp, and scanning the emission over 300–580 nm, using a 2 nm·s^−1^ scan speed and slit widths of 2 nm. The final concentration of both polypeptides was matched at 10.0 μM. Spectra were recorded in 100 mM KCl, 20 mM K_2_HPO_4_/KH_2_PO_4_ (pH 7) with or without the denaturant GdmCl present at a final concentration of 7.4 M. Assuming randomized orientations of the donor and acceptor groups and a Förster distance (*R*_*0*_) of 23.6 angstroms for the Dansyl/Trp pair, their average distance, < r > , can be estimated using the equation < r >  = $\sqrt[6]{({R}_{0}^{6}-E{R}_{0}^{6})/E}$, where E is the fraction of energy transferred from donor to acceptor [76].

A Jasco 810 spectropolarimeter fitted with a Peltier temperature control unit was used to record far UV-CD spectra at 5 and 35 °C on the complete hCPEP3 IDD at ca. 5 μM in 1 mM deuterated acetic acid (pH 4.0) using a 1.2-nm bandwidth scanning from 260 to 190 nm in a 0.1-cm quartz cuvette at 50 nm·min^−1^. Eight scans were recorded and averaged for each spectrum.

### NMR spectroscopy: instrumentation

All spectra for the hCPEB3 segments were recorded on a Bruker 800 MHz (^1^H) Avance spectrometer fitted with a triple resonance TCI cryoprobe and Z-gradients. The ^1^H chemical shift was referenced to 50 µM DSS measured in the same buffer and at the same temperatures as those used for the hCPEB3 segments. Since DSS can sometimes bind to intrinsically disordered proteins [77], the DSS signal was recorded in an independent reference tube. The ^13^C and ^15^N chemical shift references values were calculated by multiplying by their respective gyromagnetic ratios with ^1^H; that is Ξ ^13^C/Ξ ^1^H = 0.251449530 and Ξ ^15^N/Ξ ^1^H = 0.101329118 [78]. NMR spectra were recorded and transformed using TOPSPIN (versions 2.1) (Bruker Biospin).

### ^13^C-detection assignment strategy

Among the NMR approaches for studying disordered proteins (reviewed by [79]), ^13^C-detection has been gaining in popularity as it affords the characterization of proline residues [80, 81] and offers superior signal dispersion [82]. Here, to speed and improve the assignment of the backbone, we used a “proton-less” NMR approach for segments 1, 4, 5, 6, and 8 based on 2D CON spectra in which successive ^15^N-^13^CO nuclei correlations are obtained in two 3D spectra called hacacoNcaNCO and hacaCOncaNCO [83]. For segments 7 and 8, which tend to form condensates [24] and seem to be more rigid, this strategy afforded less intense spectra and in particular about 35 CON crosspeaks were missing. Therefore, an additional strategy based on ^13^CO connectivities from 3D HNCO and HNcaCO spectra as well as ^1^HN and ^15^N connectivities of consecutive residues from 3D HncocaHN and hNcocaNH spectra [84, 85] was utilized to check and complete the backbone assignments. The success of this approach seems to be due to a very slow equilibrium between aggregated protein molecules and those remaining in solution, which are detectable by NMR. The latter strategy was also employed for segment 3, which was less soluble. For all segments, further corroboration was obtained by conventional 2D ^1^H-^15^N HSQC and 3D HNCO spectra as well as 3D CCCON to confirm the residue identity and obtain the chemical shift values of ^13^C nuclei of the side chains.

Of the eight segments, only segment 2 failed to yield a soluble sample. Whereas the sequence assignments are complete thanks to the analysis of segments 1 and 3, to test for possible end effects, a 20 residue peptide, hCPEBpep2, corresponding to residues 91–110 of the hCPEB3 sequence was assigned and characterized structurally by 2D ^1^H-^1^H COSY, ^1^H-^1^H TOSCY, ^1^H-^1^H NOESY, and 2D ^1^H-^13^C HSQC NMR spectra at 5.0 °C on a Bruker 600 MHz spectrometer fitted with a cryoprobe and Z-gradients. A 2D ^1^H-^15^N HSQC was also recorded on the 800-MHz Bruker spectrometer. Both the ^1^H-^15^N HSQC and the ^1^H-^13^C HSQC spectra were recorded at the natural abundance of ^15^N and ^13^C. The program NMRFAM-Sparky [86] was used to facilitate manual spectral assignment. The NMR spectral parameters are summarized in Additional File 1. Table S1.

Theoretical chemical shift values (δ_coil_) for statistical coil ensembles were calculated using the parameters tabulated by [87] and [88], as implemented on the server at the Bax laboratory. These values were used to calculate conformational chemical shifts (Δδ), as the experimentally measured chemical shift (δ_exp_) minus the calculated chemical shift (δ_coil_). Segments of five or more residues with Δδ^13^Cα > 0.3 ppm and Δδ^13^CO > 0.3 were considered to have a significant preference to form an α-helical segment. When appropriate, families of representative preferred conformers were obtained using the program CYANA 3.98 [89] using the chemical shift data to delimit helical segments. The conformers with the lowest energy functions were chosen to be represented in the figures.

### Coupling constants

For segment 5, as an additional, independent test, a 3D HNHA spectrum were recorded and the ratio of the ^1^Hα-^1^HN crosspeak to the ^1^HN-^1^HN diagonal peak intensities in the 3D HNHA spectrum was utilized to calculate the ^3^J_HNCHα_ coupling constants following the procedure of [90]. ^3^J_HNCHα_ coupling constants were also measured for hCPEBpep2 using the 2D ^1^H-^1^H COSY spectrum. Utilizing the Karplus equation [91], these ^3^J_HNCHα_ coupling constants can be related at the backbone Φ angle, which is different for α-helical, statistical coil, and β-strands.

### Relaxation

To assess the dynamics on the ps–ns time scales, the heteronuclear ^15^N{^1^H} NOE (hNOE) of backbone amide groups was registered as the ratio of spectra recorded with and without saturation in an interleaved mode. Long recycling delays of 13 s were used. Two sets of experiments, one at 25 °C and one at 5 °C, were recorded at 800 MHz. Uncertainties in peak integrals were determined from the standard deviation of intensities from spectral regions devoid of signal which contain only noise.

In addition, R_1_ρ relaxation rates which are sensitive to the presence of preferred, rigid conformers on slower µs-ms timescales were measured by recording two sets of ten ^1^H-^15^N correlation spectra with relaxation delays at 8, 300, 36, 76, 900, 100, 500, 156, 200, and 700 ms. One set of experiments was recorded at 25 °C, and the second was recorded at 5 °C. The relaxation rates were calculated by least-squares fitting of an exponential decay function to the data using NMRPipe [92]. As an additional check, the data were also analyzed independently by using the program DynamicsCenter 2.5.2 (Bruker Biospin).

^13^C detected relaxation experiments were measured for segment 1 to confirm the presence of a rigid N-terminus and to determine proline residue imino ^15^N relaxation rates. Transverse relaxation rates (R_2_) of hCPEB3 segment 1 imino ^15^N nuclei were measured by a ^13^C-detected c_hcacon_nt2_ia3d pulse sequence [80] as a pseudo 3D experiment time composed of nine 2D experiments with relaxation delays of 15.9, 79.2, 158.4, 269.3, 396.0, 554.4, 712.8, 871.2, and 1030 ms over a ^15^ N chemical shift range that is selective for the Pro ^15^N chemical shifts (132–140 ppm). This experiment, and a similar one with a wider sweep width to also measure the ^15^N T_2_ relaxation of all 20 imino/amino-acid residues, was recorded at 25 °C, without non-uniform sampling or linear prediction. Following Fourier transformation, IPAP virtual decoupling, and baseline correction, the peaks were integrated with TOPSPIN 4.0.8 or alternatively NMRPipe by a different operator for comparison. A single exponential decay curve was then fitted to the peak integral versus time data to calculate R_2_ rates for each position.

## Supplementary Information

**Additional file 1:** **Table S1.** NMR Spectral Parameters. **Fig. S1.** Biophysical Characterization of the Complete hCPEB3 IDR. **Fig. S2.** Sequence Alignments of CPEB3 from Representative Vertebrates. **Fig. S3.** 2D ^1^H-^15^N HSQC NMR Spectrum of hCPEB3, Segment 4. **Fig. S4.** 2D ^13^CO-^15^N NMR Spectrum of hCPEB3. **Fig. S5.** ^1^H-^15^N HSQC spectra of hCPEB3 IDR Segments. **Fig. S6.** 2D ^13^CO-^15^N spectra of hCPEB3 IDR Segments. **Fig. S7.** Corroboration of Small to Negligible Populations of α-helix or β-strand Conformations in Residues 91—110 of hCPEB3. **Fig. S8.** Conformational Chemical Shifts Reveal the Presence of Partially Populated Secondary Structure in the hCPEB3 IDR. **Fig. S9.** ^13^C-detected ^15^N Relaxation Experiments of hCPEB3 segment 1. **Fig. S10.** Partial Formation of an α-Helix in the N-terminal Residues of hCPEB3. **Fig. S11.** The Consecutive Proline Residues of hCPEB3 Show a Characteristic Pattern of Conformational Chemical Shifts and Weak Binding to Profilin. **Fig. S12.** Residue Level Dynamics of hCPEB3’s Instrinsically Disordered Region. **Fig. S13. **^1^HN-^1^Hα Coupling Constants for Segment 5 Confirm the Presence of α-Helices. **Fig. S14.** Helices from Pathological and Functional Amyloids Are Stabilized by Distinct Interactions. **Fig. S15.** Phosphorylation of S224 May Increase the α-helix Population of the S224-A233 Segment. **Fig. S16.** Insight into Interhelix Interactions from Förster Resonance Energy Transfer.

## Data Availability

All data needed to evaluate the conclusions in the paper are present in the paper and/or the Supplementary Materials. All data generated or analyzed during this study are included in this published article, its supplementary information files, and publicly available repositories. In particular, NMR chemical shift data are deposited in the BMRB database under file number 50256 [93], and NMR raw spectral data are deposited in the Mendeley data repository at Laurents, Douglas; Pantoja, David (2021) “hCPEB3_NMR_Spectra” Mendeley Data V1, doi:1,017,632/hpyjdp33fx/2, at this link: https://data.mendeley.com/datasets/hpyjdp33fx/2.

## Abbreviations

*Ap*CPEB: *Aplysia* CPEB
AMPA receptors: α-Amino-3-hydroxy-5-methyl-4-isoxazolepropionic acid receptor
CPEB: Cytoplasmic Polyadenylation Element Binding protein
DSS: 4,4-Dimethyl-4-silapentane-1-sulfonic acid
FRET: Föster resonance energy transfer
GdmCl: Guanidinium chloride
HFIP: Hexafluoroisopropanol
hCPEB3: Human homolog CPEB3
IDR: Intrinsically disordered region
IPTG: Isopropyl β-D-1-thiogalactopyranoside
NLS: Nuclear localization signal
PPII: Polyproline II
PLD: Prion-like domain
RRM: RNA recognition motif
STAT5B: Signal transducer and activator of transcription 5B
TFE: Trifluoroethanol
ZnF: Zinc Finger

## References

[1] Crick F (1984). Memory and molecular turnover. Nature.

[2] Si K, Giustetto M, Etkin A, Hsu R, Janisiewicz AM, Miniaci MC, et al. A neuronal isoform of CPEB regulates local protein synthesis and stabilizes synapse-specific long-term facilitation in *Aplysia*. Cell. 2003;115:893–904. 10.1016/s0092-8674(03)01021-3 14697206

[3] Si K, Choi Y-B, White-Grindley E, Majumdar A, Kandel ER (2010). Aplysia CPEB can form prion-like multimers in sensory neurons that contribute to long-term facilitation. Cell.

[4] Raveendra BL, Siemer AB, Puthanveettil SV, Hendrickson WA, Kandel ER, McDermott AE (2013). Characterization of prion-like conformational changes of the neuronal isoform of Aplysia CPEB. Nat Struct Mol Biol.

[5] Hervas R, Rau MJ, Park Y, Zhang W, Murzin AG, Fitzpatrick JAJ (2020). Cryo-EM structure of a neuronal functional amyloid implicated in memory persistence in Drosophila. Science.

[6] Majumdar A, Cesario WC, White-Grindley E, Jiang H, Ren F, Khan MR (2012). Critical role of amyloid-like oligomers of Drosophila Orb2 in the persistence of memory. Cell.

[7] White-Grindley E, Li L, Mohammad Khan R, Ren F, Saraf A, Florens L (2014). Contribution of Orb2A stability in regulated amyloid-like oligomerization of Drosophila Orb2. PLoS Biol.

[8] Khan MR, Li L, Pérez-Sánchez C, Saraf A, Florens L, Slaughter BD (2015). Amyloidogenic oligomerization transforms drosophila Orb2 from a translation repressor to an activator. Cell.

[9] Gill J, Park Y, McGinnis JP, Perez-Sanchez C, Blanchette M, Si K (2017). Regulated intron removal integrates motivational state and experience. Cell.

[10] Hervás R, Li L, Majumdar A, Fernández-Ramírez MDC, Unruh JR, Slaughter BD (2016). Molecular basis of Orb2 amyloidogenesis and blockade of memory consolidation. PLoS Biol.

[11] Oroz J, Félix SS, Cabrita EJ, Laurents DV (2020). Structural transitions in Orb2 prion-like domain relevant for functional aggregation in memory consolidation. J Biol Chem.

[12] Fioriti L, Myers C, Huang Y-Y, Li X, Stephan JS, Trifilieff P (2015). The persistence of hippocampal-based memory requires protein synthesis mediated by the prion-like protein CPEB3. Neuron.

[13] Stephan JS, Fioriti L, Lamba N, Colnaghi L, Karl K, Derkatch IL (2015). The CPEB3 protein is a functional prion that interacts with the actin cytoskeleton. Cell Rep.

[14] Drisaldi B, Colnaghi L, Fioriti L, Rao N, Myers C, Snyder AM (2015). SUMOylation is an inhibitory constraint that regulates the prion-like aggregation and activity of CPEB3. Cell Rep.

[15] Chao H-W, Lai Y-T, Lu Y-L, Lin C, Mai W, Huang Y-S (2012). NMDAR signaling facilitates the IPO5-mediated nuclear import of CPEB3. Nucleic Acids Res.

[16] Terlau H, Seifert W (1990). Fibroblast growth factor enhances long-term potentiation in the hippocampal slice. Eur J Neurosci.

[17] Peng S-C, Lai Y-T, Huang H-Y, Huang H-D, Huang Y-S (2010). A novel role of CPEB3 in regulating EGFR gene transcription via association with Stat5b in neurons. Nucleic Acids Res.

[18] Tsuda K, Kuwasako K, Nagata T, Takahashi M, Kigawa T, Kobayashi N (2014). Novel RNA recognition motif domain in the cytoplasmic polyadenylation element binding protein 3. Proteins.

[19] Huang Y-S, Kan M-C, Lin C-L, Richter JD (2006). CPEB3 and CPEB4 in neurons: analysis of RNA-binding specificity and translational control of AMPA receptor GluR2 mRNA. EMBO J.

[20] Ford L, Ling E, Kandel ER, Fioriti L (2019). CPEB3 inhibits translation of mRNA targets by localizing them to P bodies. Proc Natl Acad Sci U S A.

[21] Cougot N, Bhattacharyya SN, Tapia-Arancibia L, Bordonné R, Filipowicz W, Bertrand E (2008). Dendrites of mammalian neurons contain specialized P-body-like structures that respond to neuronal activation. J Neurosci.

[22] Kandel ER, Dudai Y, Mayford MR (2014). The molecular and systems biology of memory. Cell.

[23] Vogler C, Spalek K, Aerni A, Demougin P, Müller A, Huynh K-D (2009). CPEB3 is associated with human episodic memory. Front Behav Neurosci.

[24] Ramírez de Mingo D, López-García P, Hervás R, Laurents DV, Carrión-Vázquez M. Molecular determinants of liquid demixing and amyloidogenesis in human CPEB3. bioRxiv. 2020:2020.06.02.129783.

[25] Jumper J, Evans R, Pritzel A, Green T, Figurnov M, Ronneberger O (2021). Highly accurate protein structure prediction with AlphaFold. Nature.

[26] Alston RW, Urbanikova L, Sevcik J, Lasagna M, Reinhart GD, Scholtz JM (2004). Contribution of single tryptophan residues to the fluorescence and stability of ribonuclease Sa. Biophys J.

[27] Denning DP, Patel SS, Uversky V, Fink AL, Rexach M (2003). Disorder in the nuclear pore complex: the FG repeat regions of nucleoporins are natively unfolded. Proc Natl Acad Sci U S A.

[28] Bundi A, Wüthrich K. ^1^H-NMR parameters of the common amino acid residues measured in aqueous solution of the linear tetrapeptides H-Gly-Gly-X-L-Ala-OH. Biopolymers. 1979;18:285–97.

[29] López-Alonso JP, Bruix M, Font J, Ribó M, Vilanova M, Jiménez MA (2010). NMR spectroscopy reveals that RNase A is chiefly denatured in 40% acetic acid: implications for oligomer formation by 3D domain swapping. J Am Chem Soc.

[30] Mukrasch MD, Bibow S, Korukottu J, Jeganathan S, Biernat J, Griesinger C (2009). Structural polymorphism of 441-residue tau at single residue resolution. PLoS Biol.

[31] Muñoz V, Serrano L, Jiménez MA, Rico M (1995). Structural analysis of peptides encompassing all alpha-helices of three alpha/beta parallel proteins: Che-Y, flavodoxin and P21-ras: implications for alpha-helix stability and the folding of alpha/beta parallel proteins. J Mol Biol.

[32] Bhattacharyya A, Thakur AK, Chellgren VM, Thiagarajan G, Williams AD, Chellgren BW (2006). Oligoproline effects on polyglutamine conformation and aggregation. J Mol Biol.

[33] Treviño MÁ, Pantoja-Uceda D, Menéndez M, Gomez MV, Mompeán M, Laurents DV (2018). The singular NMR fingerprint of a polyproline II helical bundle. J Am Chem Soc.

[34] Spera S, Bax A. Empirical correlation between protein backbone conformation and Calpha and Cbeta ^13^C NMR chemical shifts. J Am Chem Soc. 1991;113:5490–2.

[35] Wang Y, Jardetzky O (2002). Probability-based protein secondary structure identification using combined NMR chemical-shift data. Protein Sci.

[36] Wishart DS, Sykes BD. The ^13^C chemical-shift index: a simple method for the identification of protein secondary structure using ^13^C chemical-shift data. J Biomol NMR. 1994;4:171–80. 10.1007/BF00175245 8019132

[37] Reselammal DS, Pinhero F, Sharma R, Oliyantakath Hassan MS, Srinivasula SM, Vijayan V (2021). Mapping the fibril core of the prion subdomain of the mammalian CPEB3 that is involved in long term memory retention. J Mol Biol.

[38] Kelly MA, Chellgren BW, Rucker AL, Troutman JM, Fried MG, Miller AF (2001). Host-guest study of left-handed polyproline II helix formation. Biochemistry.

[39] Metzler WJ, Bell AJ, Ernst E, Lavoie TB, Mueller L (1994). Identification of the poly-L-proline-binding site on human profilin. J Biol Chem.

[40] Petrella EC, Machesky LM, Kaiser DA, Pollard TD (1996). Structural requirements and thermodynamics of the interaction of proline peptides with profilin. Biochemistry.

[41] Aurora R, Rose GD (1998). Helix capping. Protein Sci.

[42] Chakrabartty A, Kortemme T, Baldwin RL (1994). Helix propensities of the amino acids measured in alanine-based peptides without helix-stabilizing side-chain interactions. Protein Sci.

[43] Chakrabartty A, Baldwin RL (1995). Stability of alpha-helices. Adv Protein Chem.

[44] Andrew CD, Warwicker J, Jones GR, Doig AJ (2002). Effect of phosphorylation on alpha-helical stability as a function of position. Biochemistry.

[45] Morón-Oset J, Supèr T, Esser J, Isaacs AM, Grönke S, Partridge L (2019). Glycine-alanine dipeptide repeats spread rapidly in a repeat length- and age-dependent manner in the fly brain. Acta Neuropathol Commun.

[46] Mizielinska S, Grönke S, Niccoli T, Ridler CE, Clayton EL, Devoy A (2014). C9orf72 repeat expansions cause neurodegeneration in Drosophila through arginine-rich proteins. Science.

[47] Kaczmarczyk L, Labrie-Dion É, Sehgal K, Sylvester M, Skubal M, Josten M (2016). New Phosphospecific antibody reveals isoform-specific phosphorylation of CPEB3 protein. PLoS ONE.

[48] Chong PA, Vernon RM, Forman-Kay JD (2018). RGG/RG motif regions in RNA binding and phase separation. J Mol Biol.

[49] Brown CJ, Takayama S, Campen AM, Vise P, Marshall TW, Oldfield CJ (2002). Evolutionary rate heterogeneity in proteins with long disordered regions. J Mol Evol.

[50] Escobedo A, Topal B, Kunze MBA, Aranda J, Chiesa G, Mungianu D (2019). Side chain to main chain hydrogen bonds stabilize a polyglutamine helix in a transcription factor. Nat Commun.

[51] Cao Q, Boyer DR, Sawaya MR, Ge P, Eisenberg DS (2019). Cryo-EM structures of four polymorphic TDP-43 amyloid cores. Nat Struct Mol Biol.

[52] Arseni D, Hasegawa M, Murzin AG, Kametani F, Arai M, Yoshida M (2021). Structure of pathological TDP-43 filaments from ALS with FTLD. Nature.

[53] Pentelute BL, Gates ZP, Tereshko V, Dashnau JL, Vanderkooi JM, Kossiakoff AA (2008). X-ray structure of snow flea antifreeze protein determined by racemic crystallization of synthetic protein enantiomers. J Am Chem Soc.

[54] Guo YE, Manteiga JC, Henninger JE, Sabari BR, Dall’Agnese A, Hannett NM (2019). Pol II phosphorylation regulates a switch between transcriptional and splicing condensates. Nature.

[55] Li H-R, Chiang W-C, Chou P-C, Wang W-J, Huang J-R (2018). TAR DNA-binding protein 43 (TDP-43) liquid-liquid phase separation is mediated by just a few aromatic residues. J Biol Chem.

[56] Fiumara F, Fioriti L, Kandel ER, Hendrickson WA (2010). Essential role of coiled coils for aggregation and activity of Q/N-rich prions and PolyQ proteins. Cell.

[57] Pelassa I, Corà D, Cesano F, Monje FJ, Montarolo PG, Fiumara F (2014). Association of polyalanine and polyglutamine coiled coils mediates expansion disease-related protein aggregation and dysfunction. Hum Mol Genet.

[58] Lilliu E, Villeri V, Pelassa I, Cesano F, Scarano D, Fiumara F (2018). Polyserine repeats promote coiled coil-mediated fibril formation and length-dependent protein aggregation. J Struct Biol.

[59] Polling S, Ormsby AR, Wood RJ, Lee K, Shoubridge C, Hughes JN (2015). Polyalanine expansions drive a shift into α-helical clusters without amyloid-fibril formation. Nat Struct Mol Biol.

[60] Hervás R, Del Carmen F-R, Galera-Prat A, Suzuki M, Nagai Y, Bruix M (2021). Divergent CPEB prion-like domains reveal different assembly mechanisms for a generic amyloid-like fold. BMC Biol.

[61] Graether SP, Slupsky CM, Sykes BD (2003). Freezing of a fish antifreeze protein results in amyloid fibril formation. Biophys J.

[62] Hamley IW, Kirkham S, Dehsorkhi A, Castelletto V, Adamcik J, Mezzenga R (2014). Self-assembly of a model peptide incorporating a hexa-histidine sequence attached to an oligo-alanine sequence, and binding to gold NTA/nickel nanoparticles. Biomacromol.

[63] Albrecht A, Mundlos S (2005). The other trinucleotide repeat: polyalanine expansion disorders. Curr Opin Genet Dev.

[64] Ferron F, Rebowski G, Lee SH, Dominguez R (2007). Structural basis for the recruitment of profilin-actin complexes during filament elongation by Ena/VASP. EMBO J.

[65] Basu S, Lamprecht R (2018). The role of actin cytoskeleton in dendritic spines in the maintenance of long-term memory. Front Mol Neurosci.

[66] Salehi-Ashtiani K, Lupták A, Litovchick A, Szostak JW (2006). A genomewide search for ribozymes reveals an HDV-like sequence in the human CPEB3 gene. Science.

[67] Huang W-H, Chao H-W, Tsai L-Y, Chung M-H, Huang Y-S (2014). Elevated activation of CaMKIIα in the CPEB3-knockout hippocampus impairs a specific form of NMDAR-dependent synaptic depotentiation. Front Cell Neurosci.

[68] Marley J, Lu M, Bracken C (2001). A method for efficient isotopic labeling of recombinant proteins. J Biomol NMR.

[69] Nallamsetty S, Kapust RB, Tözsér J, Cherry S, Tropea JE, Copeland TD, et al. Efficient site-specific processing of fusion proteins by tobacco vein mottling virus protease *in vivo* and *in vitro*. Protein Expr Purif. 2004;38:108–15. 10.1016/j.pep.2004.08.016 15477088

[70] Li M, Liu J, Ran X, Fang M, Shi J, Qin H (2006). Resurrecting abandoned proteins with pure water: CD and NMR studies of protein fragments solubilized in salt-free water. Biophys J.

[71] Mompeán M, Romano V, Pantoja-Uceda D, Stuani C, Baralle FE, Buratti E (2016). The TDP-43 N-terminal domain structure at high resolution. FEBS J.

[72] Chaves-Arquero B, Pérez-Cañadillas JM, Jiménez MA (2020). Effect of phosphorylation on the structural behaviour of peptides derived from the intrinsically disordered C-terminal domain of histone H1.0. Chemistry.

[73] Borgia A, Borgia MB, Bugge K, Kissling VM, Heidarsson PO, Fernandes CB (2018). Extreme disorder in an ultrahigh-affinity protein complex. Nature.

[74] Di Tommaso P, Moretti S, Xenarios I, Orobitg M, Montanyola A, Chang JM (2011). T-Coffee: a web server for the multiple sequence alignment of protein and RNA sequences using structural information and homology extension. Nucleic Acids Res.

[75] Sievers F, Wilm A, Dineen D, Gibson TJ, Karplus K, Li W (2011). Fast, scalable generation of high-quality protein multiple sequence alignments using Clustal Omega. Mol Syst Biol.

[76] Lakowicz JR, Gryczynski I, Wiczk W, Laczko G, Prendergast FC, Johnson ML (1990). Conformational distributions of melittin in water/methanol mixtures from frequency-domain measurements of nonradiative energy transfer. Biophys Chem.

[77] Diez-García F, Gómez-Pinto I, Chakrabartty A, González C, Laurents DV (2012). Conformation specificity and arene binding in a peptide composed only of Lys, Ile. Ala and Gly Eur Biophys J.

[78] Markley JL, Bax A, Arata Y, Hilbers CW, Kaptein R, Sykes BD (1998). Recommendations for the presentation of NMR structures of proteins and nucleic acids–IUPAC-IUBMB-IUPAB Inter-Union Task Group on the standardization of data bases of protein and nucleic acid structures determined by NMR spectroscopy. Eur J Biochem.

[79] Gibbs EB, Cook EC, Showalter SA (2017). Application of NMR to studies of intrinsically disordered proteins. Arch Biochem Biophys.

[80] Murrali MG, Piai A, Bermel W, Felli IC, Pierattelli R (2018). Proline fingerprint in intrinsically disordered proteins. ChemBioChem.

[81] Mateos B, Conrad-Billroth C, Schiavina M, Beier A, Kontaxis G, Konrat R (2020). The ambivalent role of proline residues in an intrinsically disordered protein: from disorder promoters to compaction facilitators. J Mol Biol.

[82] Cook EC, Usher GA, Showalter SA (2018). The use of (13)C Direct-Detect NMR to characterize flexible and disordered proteins. Methods Enzymol.

[83] Pantoja-Uceda D, Santoro J. New ^13^C-detected experiments for the assignment of intrinsically disordered proteins. J Biomol NMR. 2014;59:43–50. 10.1007/s10858-014-9827-1 24699834

[84] Sun ZYJ, Frueh DP, Selenko P, Hoch JC, Wagner G. Fast assignment of ^15^N-HSQC peaks using high-resolution 3D HNcocaNH experiments with non-uniform sampling. J Biomol NMR. 2005;33:43–50. 10.1007/s10858-005-1284-4 16222556

[85] Pantoja-Uceda D, Santoro J (2009). Aliasing in reduced dimensionality NMR spectra: (3,2)D HNHA and (4,2)D HN(COCA)NH experiments as examples. J Biomol NMR.

[86] Lee W, Tonelli M, Markley JL (2015). NMRFAM-SPARKY: enhanced software for biomolecular NMR spectroscopy. Bioinformatics.

[87] Kjaergaard M, Poulsen FM (2011). Sequence correction of random coil chemical shifts: correlation between neighbor correction factors and changes in the Ramachandran distribution. J Biomol NMR.

[88] Kjaergaard M, Brander S, Poulsen FM (2011). Random coil chemical shift for intrinsically disordered proteins: effects of temperature and pH. J Biomol NMR.

[89] Güntert P, Buchner L (2015). Combined automated NOE assignment and structure calculation with CYANA. J Biomol NMR.

[90] Vuister GW, Bax A. Quantitative J correlation: a new approach for measuring homonuclear J(HN-Ha) coupling constants in ^15^N-enriched proteins. J Am Chem Soc. 1993;115:7772–7.

[91] Karplus M (1963). Vicinal proton coupling in nuclear magnetic resonance. J Am Chem Soc.

[92] Delaglio F, Grzesiek S, Vuister GW, Zhu G, Pfeifer J, Bax A (1995). NMRPipe: a multidimensional spectral processing system based on UNIX pipes. J Biomol NMR.

[93] Ulrich EL, Akutsu H, Doreleijers JF, Harano Y, Ioannidis YE, Lin J (2008). BioMagResBank. Nucleic Acids Res.

