# Supplementary material for: Conformational dynamics in the disordered region of human CPEB3 linked to memory consolidation
Source: BMC Biol. 2022 Jun 3;20:129. doi: 10.1186/s12915-022-01310-6 (PMC9166367; doi:10.1186/s12915-022-01310-6)
Supplement: Supplementary file 1 — Additional file 1: Table S1. NMR Spectral Parameters. Fig. S1. Biophysical Characterization of the Complete hCPEB3 IDR. Fig. S2. Sequence Alignments of CPEB3 from Representative Vertebrates. Fig. S3. 2D 1H-15N HSQC NMR Spectrum of hCPEB3, Segment 4. Fig. S4. 2D 13CO-15N NMR Spectrum of hCPEB3. Fig. S5. 1H-15N HSQC spectra of hCPEB3 IDR Segments. Fig. S6. 2D 13CO-15N spectra of hCPEB3 IDR Segments. Fig. S7. Corroboration of Small to Negligible Populations of α-helix or β-strand Conformations in Residues 91—110 of hCPEB3. Fig. S8. Conformational Chemical Shifts Reveal the Presence of Partially Populated Secondary Structure in the hCPEB3 IDR. Fig. S9. 13C-detected 15N Relaxation Experiments of hCPEB3 segment 1. Fig. S10. Partial Formation of an α-Helix in the N-terminal Residues of hCPEB3. Fig. S11. The Consecutive Proline Residues of hCPEB3 Show a Characteristic Pattern of Conformational Chemical Shifts and Weak Binding to Profilin. Fig. S12. Residue Level Dynamics of hCPEB3’s Instrinsically Disordered Region. Fig. S13. 1HN-1Hα Coupling Constants for Segment 5 Confirm the Presence of α-Helices. Fig. S14. Helices from Pathological and Functional Amyloids Are Stabilized by Distinct Interactions. Fig. S15. Phosphorylation of S224 May Increase the α-helix Population of the S224-A233 Segment. Fig. S16. Insight into Interhelix Interactions from Förster Resonance Energy Transfer. [file 12915_2022_1310_MOESM1_ESM.docx]

**Additional File 1**

**Supporting Information for:**

**Conformational dynamics in the disordered region of human CPEB3,**

**linked to memory consolidation**

D. Ramírez de Mingo,^1^ D. Pantoja-Uceda,^2^ R. Hervás,^3^

M. Carrión-Vázquez,^1*^ D. V. Laurents, ^2*^

^1^Instituto Cajal, IC-CSIC, Avda. Doctor Arce 37, E-28002 Madrid, Spain.

^2^Instituto de Química-Física Rocasolano, IQFR-CSIC, Serrano 119, E-28006 Madrid, Spain.

^3^School of Biomedical Sciences, Li Ka Shing Faculty of Medicine,

The University of Hong Kong, Pokfulam, Hong Kong, HKSAR, China.

^†^To whom correspondence should be addressed:

MCV ([mcarrion@cajal.csic.es](mailto:mcarrion@cajal.csic.es)), DVL ([dlaurents@iqfr.csic.es](mailto:dlaurents@iqfr.csic.es), @DouglasLaurents)

**Table S1:** NMR Spectral Parameters

| **Experiment** | **Number of Scans** | **Sweep Width (ppm)** | **Matrix** |
| --- | --- | --- | --- |
| *Segments 1, 3, 4, 5, 6, 7 & 8* | | | |
| 1D ^1^H | 8 | 10 | 32k |
| 2D ^1^H-^15^N HSQC | 2-8 | 10 ^1^H x 20 ^15^N | 2k x 512 |
| 2D CON^*^ | 4–16 | 10 ^13^CO x 35 ^15^N | 1k x 512 |
| 3D HNCO | 4–8 | 10 ^1^H x 20 ^15^N x 10 ^13^C | 2k x 64 x 128 |
| 3D hacacoNcaNCO^*^ | 8 | 10 ^13^CO x 35 ^15^N x 20 ^15^N | 1k x 48 x 96 |
| 3D hacaCOncaNCO^*^ | 8 | 10 ^13^CO x 35 ^15^N x 20 ^13^CO | 1k x 48 x 96 |
| 3D CCCON^*^ |  | 10 ^13^CO x 35 ^15^N x 60 ^13^C(aliphatic) | 1k x 56 x 96 |
| 3D CBCACON | 8 | 10 ^13^CO x 35 ^15^N x 60 ^13^C(aliphatic) | 1k x 64 x 80 |
| 3D HNHA^§^ | 8 | 11^1^HN x 23 ^15^N x 11 ^1^Hα | 2k x 96 x 48 |
| *Segments 3, 7 & 8*^†^ | | | |
| 3D HNcaCO­­­ | 16 | 10 ^1^H x 20 ^15^N x 10 ^13^C | 2k x 32 x 64 |
| 3D hNcocaNH | 12 | 10 ^1^H x 20 ^15^N x 20 ^15^N | 2k x 64 x 90 |
| 3D HncocaNH | 12 | 10 ^1^H x 20 ^15^N x 10 ^1^H | 2k x 96 x 48 |
| 3D CBCAcoNH | 16 | 10 ^1^H x 20 ^15^N x 10 ^13^C | 2k x 32 x 64 |
| *Relaxation* | | | |
| 2D {^1^H}-^15^N NOE^¶^ | 8-16 | 10 ^1^H x 20 ^15^N | 2k x 512 |
| 2D ^1^H-^15^N T_1ρ_^‡^ | 8-16 | 10 ^1^H x 20 ^15^N | 2k x 256 |
| 2D c_hcacon_nt2_ia3d | 4 | 10 ^13^CO x 33 ^15^N | 700 x 256 |
| 2D c_hcacon_nt2_ia3d | 8 | 10 ^13^CO x 6 ^15^N (Pro focussed) | 1k x 48 |
| *Peptides* | | | |
| 2D ^1^H-^1^H COSY | 16 | 11 x 11 | 2k x 512 |
| 2D ^1^H-^1^H TOCSY | 8 | 11 x 11, t_mix_ = 60 ms | 2k x 512 |
| 2D ^1^H-^1^H NOESY | 64 | 11 x 11, t_mix_=150 ms | 2k x 512 |
| 2D ^1^H-^13^C HSQC | 144 | 10 x 80, recorded in 100% D_2_O | 1k x 128 |
| 2D ^1^H-^15^N HSQC^**^ | 512 | 10 x 20 | 1k x 96 |

* ^13^C detection; processed with in-phase anti-phase (IPAP) virtual decoupling.

§ Recorded for some segments to measure ^1^HN-^1^Hα coupling constants.

† Recorded for segments 3, 7 and 8, whose lower sample concentration required more sensitive ^1^H-detected experiments.

¶ A delay of 10 seconds between pulses was used. Recorded in interleaved mode.

‡ Ten spectra with delays of 8, 300, 36, 76, 900, 100, 500, 156, 200 and 16 ms were recorded.

^**^ Recorded only for hCPEB3pep1 and hCPEB3pep2

**Fig. S1**

Biophysical Characterization of the Complete hCPEB3 IDR


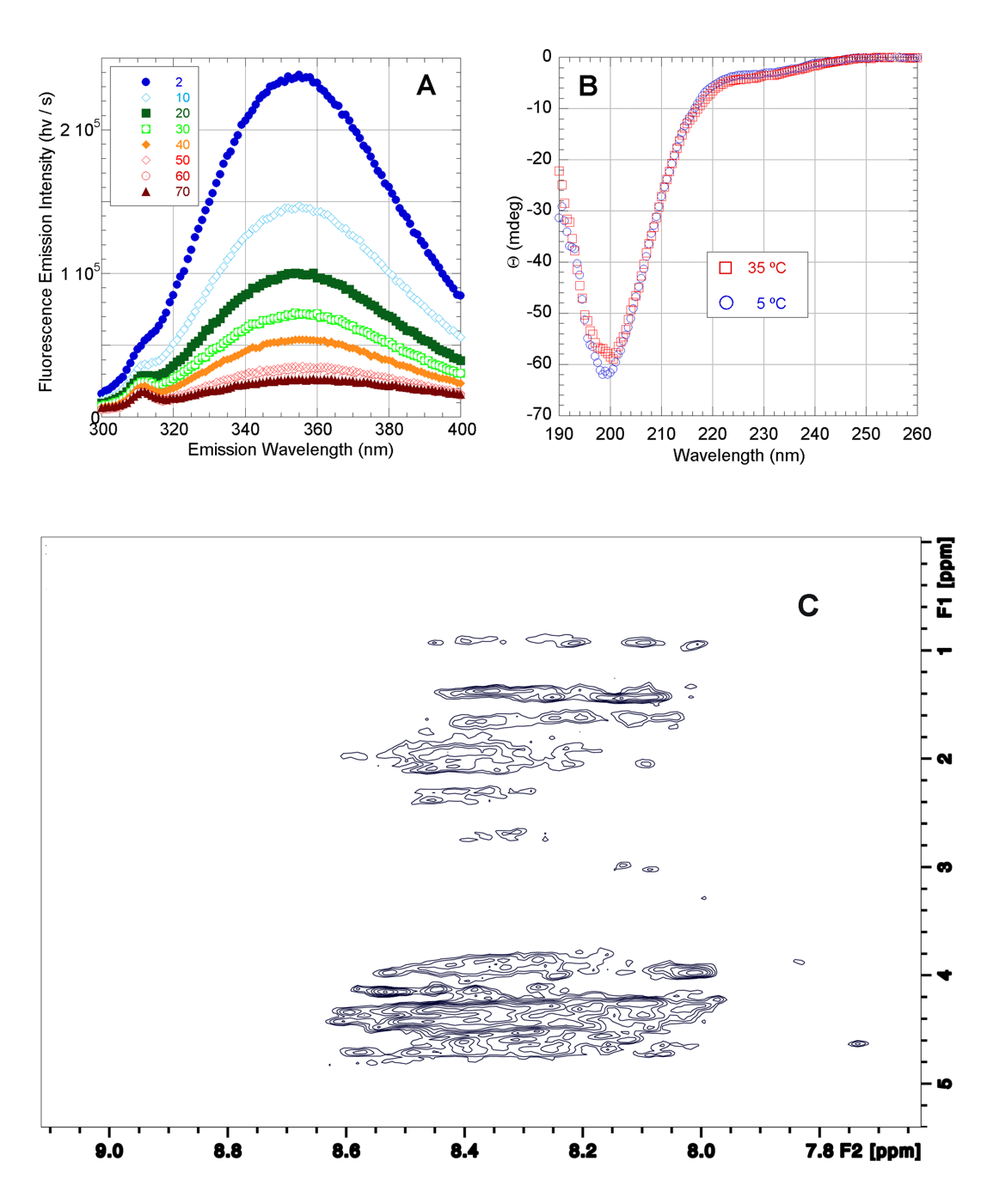


**A.** Fluorescence emission spectra of the complete IDR of hCPEB3 at pH 4.0 in 1.0 mM deuterated acetic acid buffer over temperatures ranging from 2 ºC to 70 ºC as indicated. The emission maximum over 350 nm is indicative of solvent exposed Trp side chains. This is consistent with the lack of a well packed hydrophobic core.

**B.** Far UV-CD spectra of the IDR of hCPEB3 at 5 ºC (blue open circles) and 35 ºC (red open squares). The observed minimum near 200 nm and the lack of minima near 208 nm, 218 nm and 222 nm and the lack of a maximum at 195 nm are all characteristic spectral features of a statistical coil.

**C**. 2D ^1^H-^1^H NOESY spectrum of the hCPEB3 IDR recorded at 25 ºC showing the ^1^HN crosspeak region. The small chemical shift dispersion in ^1^HN (8.6 to 7.9 ppm) is a typical feature of disordered and unfolded proteins [27].

**Fig. S2**: Sequence Alignments of CPEB3 from Representative Vertebrates

**
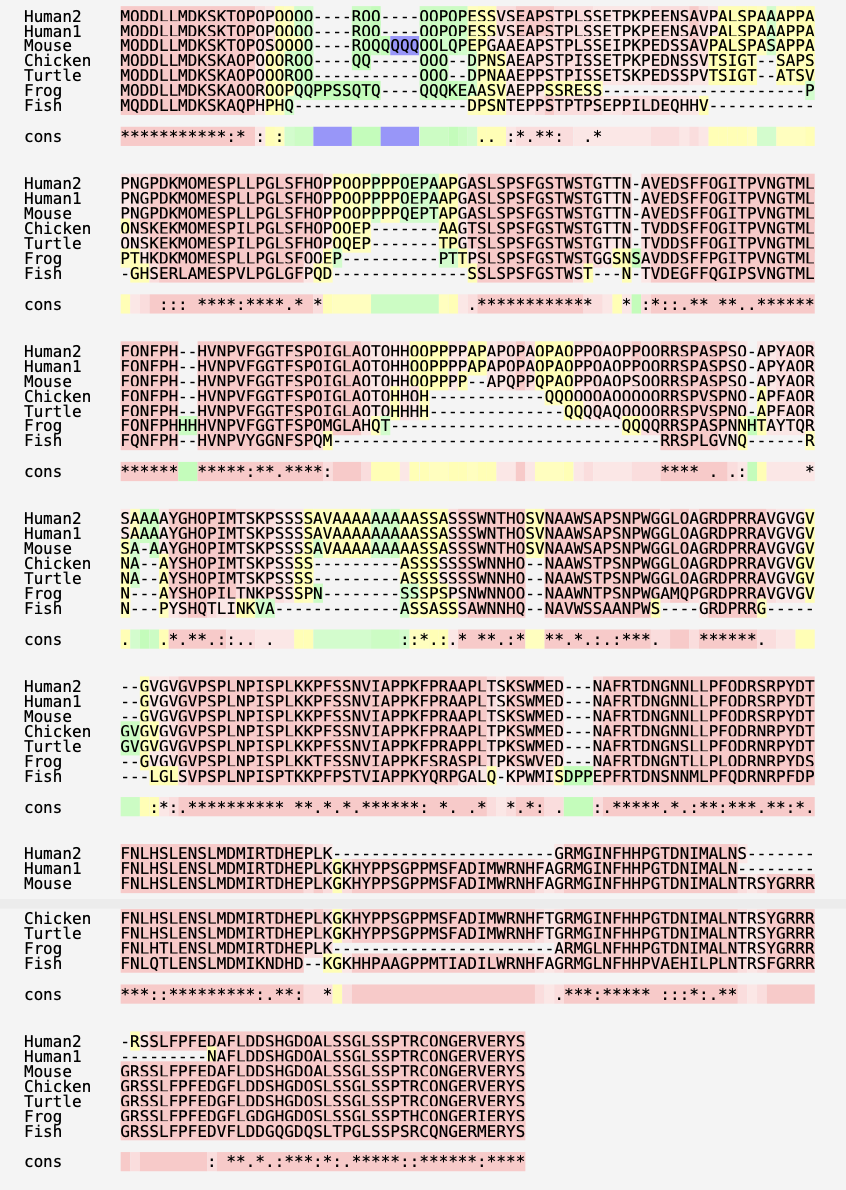
**

Results obtained using T-coffee, version 11 (Di Tommaso *et al.,* 2011) [69] using default settings. Sequence is highlighted using a red to blue color scale for highly to poorly conserved residues, respectively. Note that because of the small space between lines, some “Q”s appear as “O”s. “Cons” indicates the level of sequence conservation: “*” = strictly conserved, “:” = well conserved, “.” moderately conserved.

**Fig. S3** 2D ^1^H-^15^N HSQC NMR Spectrum of hCPEB3, Segment 4

^1^H-^15^N correlations were recorded at 25ºC in 1 mM acetic acid, pH 4. Note the cluster of Ala signals in belonging to the polyA stretches near 8.05 ppm ^1^H and 122.5 ppm ^15^N. Their position contrasts with those of the isolated Ala residues, whose δ ^15^N > 125 ppm. Signals labeled in lower case with negative numbers correspond the His/TEV tag: M_-29_GSSHHHHHHSSGLVPRGSHMASENLYFQ_-1_

**Fig. S4** 2D ^13^CO-^15^N NMR Spectrum of hCPEB3

^13^CO-^15^N correlations were recorded at 25ºC in 1 mM acetic acid, pH 4. Here, the signals of the long polyAla stretch, which appears around 122.5 ppm for ^15^N x 178.5 ppm for ^13^CO shows superior resolution relative to the ^1^H-^15^N HSQC spectrum. Their position at higher ^13^CO and lower ^15^N chemical shift values relative to isolated Ala and to coil values indicates an α-helical conformation. For simplicity, the label refers to the ^13^CO of the *i-1* residue. Signals labeled in lower case with negative numbers correspond the His/TEV tag: M_-29_GSSHHHHHHSSGLVPRGSHMASENLYFQ_-1_

**Fig. S5** ^1^H-^15^N HSQC spectra of hCPEB3 IDR Segments

2D ^1^H-^15^N HSQC spectra of hCPEB3 IDR recorded at pH 4, 25ºC on:

A. Segment 1 (**purple**), signals from the polyQ rich segment are circled.

B. Segments 3 (**blue**) and 4 (**green**). Signals from the polyA rich segment are boxed.

C. Segments 4 (**green**) and 5 (**brown**). Signals from the polyA rich segment are boxed.

D. Segments 5 (**brown**) and 6 (**orange**).

E. Segments 6 (**orange**) and 7 (**red**).

F. Segments 7 (**red**) and 8 (**black**).

**Fig. S6** 2D ^13^CO-^15^N spectra of hCPEB3 IDR Segments

**
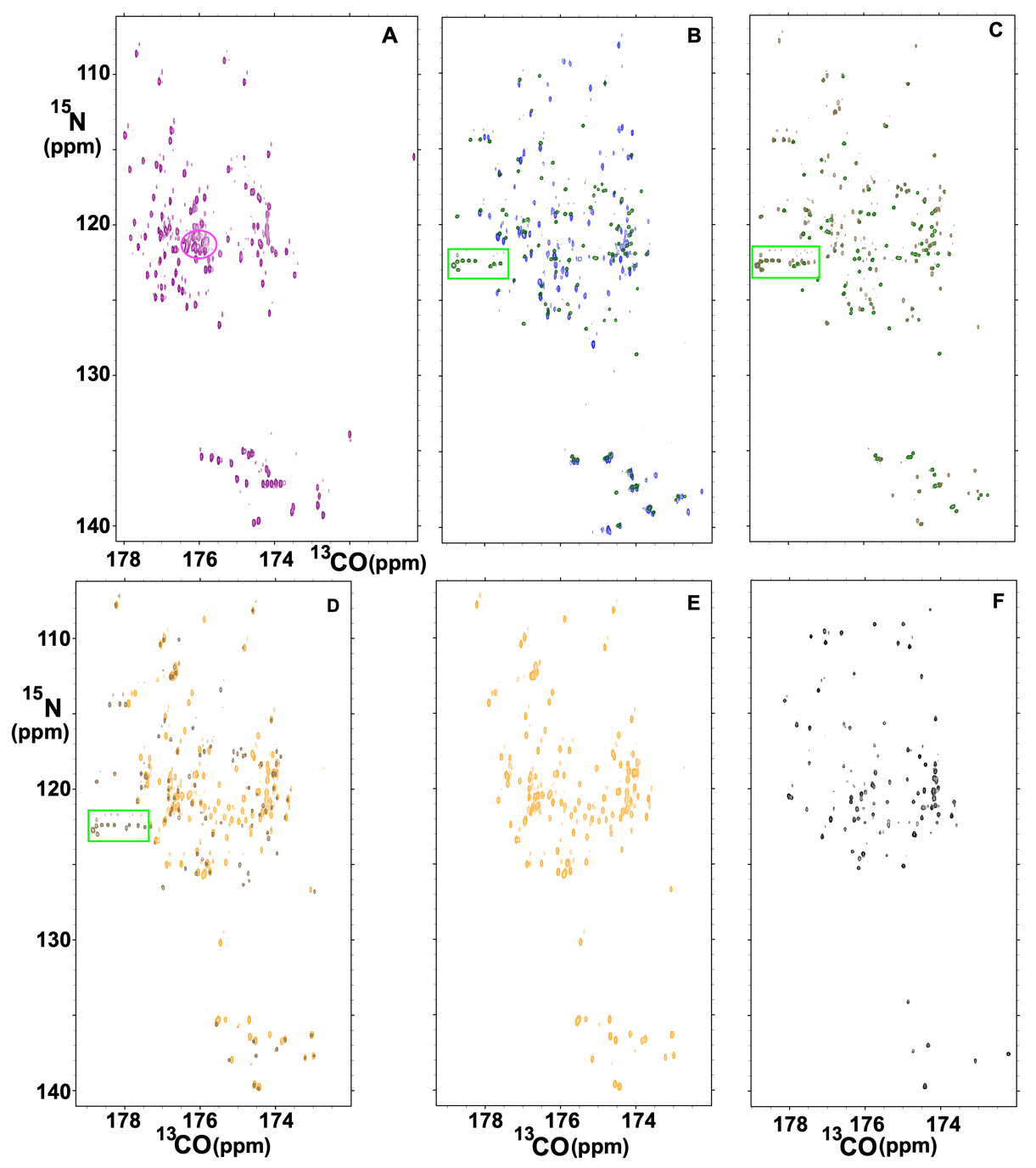
**

2D ^13^CO-^15^N HSQC spectra of hCPEB3 IDR recorded at pH 4, 25ºC on:

A. Segment 1 (**purple**), signals from the polyQ rich segment are circled in magenta.

B. Segments 3 (**blue**) and 4 (**green**). Signals from the polyA rich segment are boxed in **light green**.

C. Segments 4 (**green**) and 5 (**brown**). Signals from the polyA rich segment are boxed in **light green**.

D. Segments 5 (**brown**) and 6 (**orange**).

E. Segment 6 (**orange**).

F. Segment 8 (**black**)

The spectrum of segment 1 is displaced because its optimal spectral range was distinct relative to the other spectra. The weak satellite peaks appearing to the right and above the main peaks arise from deuteration.

**Fig. S7**. Corroboration of small to negligible populations of α-helix or β-strand conformations in residues 91 - 110 of hCPEB3.


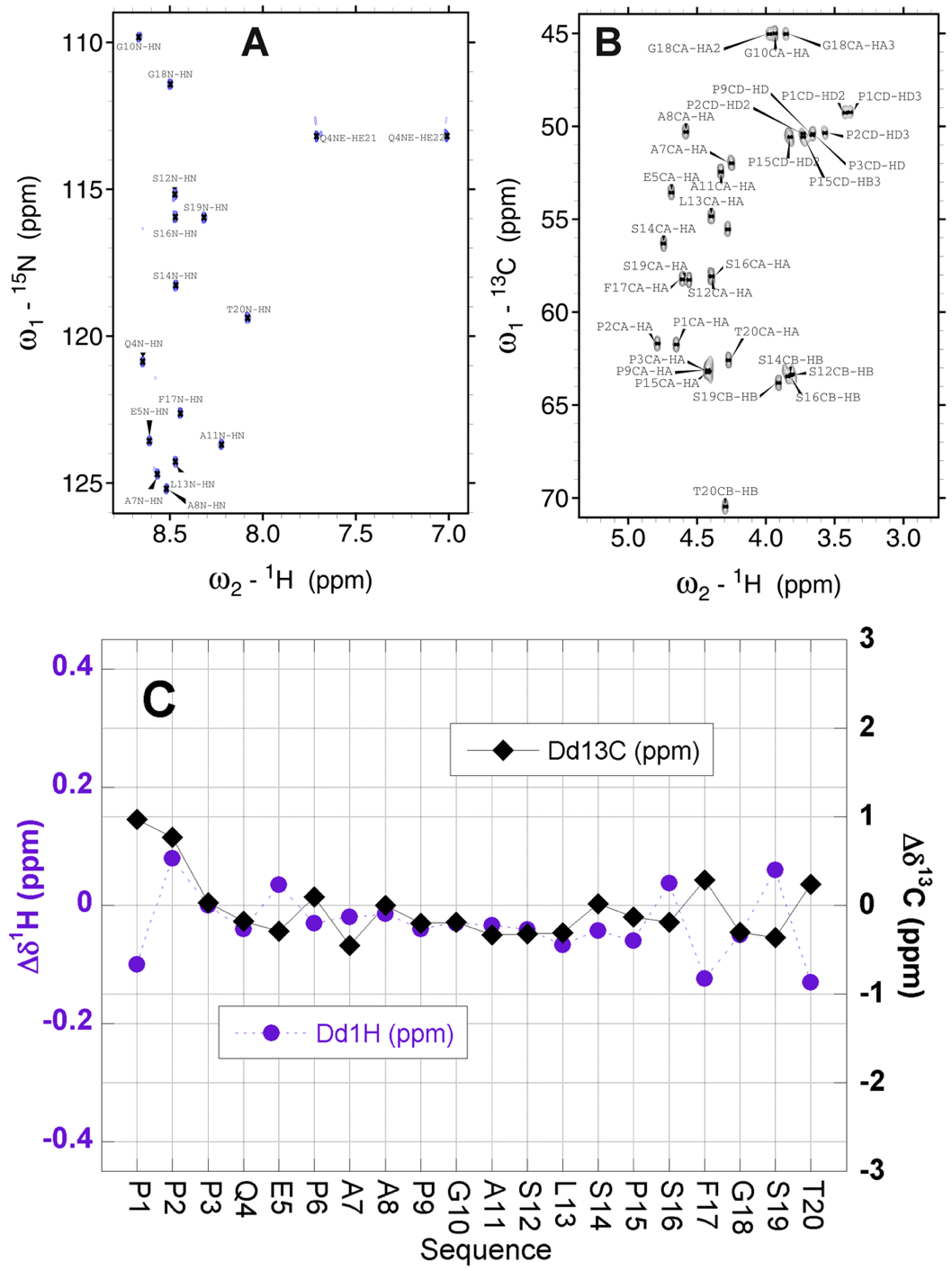


**A**. The assigned ^1^H-^15^N HSQC spectrum of a peptide corresponding to residues 91 - 110 of hCPEB3. The low chemical shift dispersion in the ^1^HN dimension is a hallmark of a statistical coil. The residue number labels correspond to this peptide, to calculate the residue number in the full length protein, add 90.

**B**. The ^1^H-^13^C HSQC spectrum of the same peptide, showing the assigned ^1^Hα-^13^Cα, ^1^Hδ-^13^Cδ Pro and ^1^Hβ-^13^Cβ Ser & Thr correlations.

**C**. Conformational chemical shifts of ^1^Hα (left y-axis, **purple circles**) and ^13^Cα (right y-axis, **black diamonds**). Values of -0.41 ppm (^1^H) and +3.1 ppm (^13^C) correspond to 100% α-helix and values of +0.41 (^1^H) and -3.1 ppm (^13^C) correspond to 100% β-strand. Here residues show small conformational chemical shifts except for N- and C-termini, whose values are perturbed by end effects, and residues near Phe17 (Phe 107 in the full length protein) whose values are altered by ring current effects.

**Fig. S8**. Conformational chemical shifts reveal the presence of partially populated secondary structure in the hCPEB3 IDR.


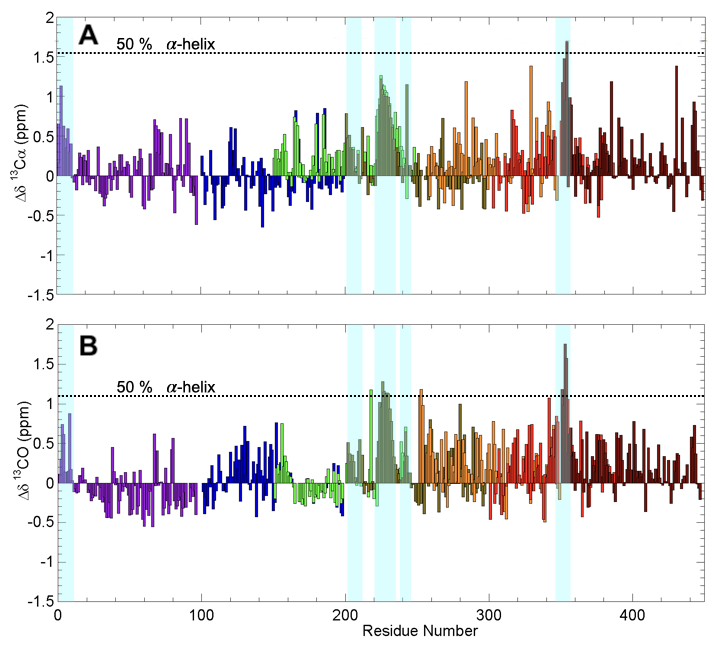


Conformational chemical shifts (Δδ) at 25ºC, calculated as the experimentally measured chemical shift (δ_exp_) minus the chemical shift expected for statistical coil (δ_coil_) for ^13^Cα (*top panel*) and ^13^CO (*bottom panel*) under these conditions. Data are colored according to segment: 1=**purple**, 3=**blue**, 4=**green**, 5=**brown**, 6=**orange**, 7=**red**, 8=**maroon**. Negative values Δδ values of ^13^Cα and ^13^CO are characteristic of extended conformations like β-strands. Values of +1.55 ppm for Δδ ^13^Cα and +1.1 ppm Δδ ^13^CO are expected for 50% α-helix; these values represented as dotted lines. Stretches of positive Δδ for ^13^Cα and Δδ ^13^CO values which evince partial α-helix formation span residues 1-10, 202-210, 222-234, 238-246 and 346-356 and are shaded cyan.

**Fig. S9** ^13^C-detected ^15^N Relaxation Experiments of hCPEB3 segment 1.


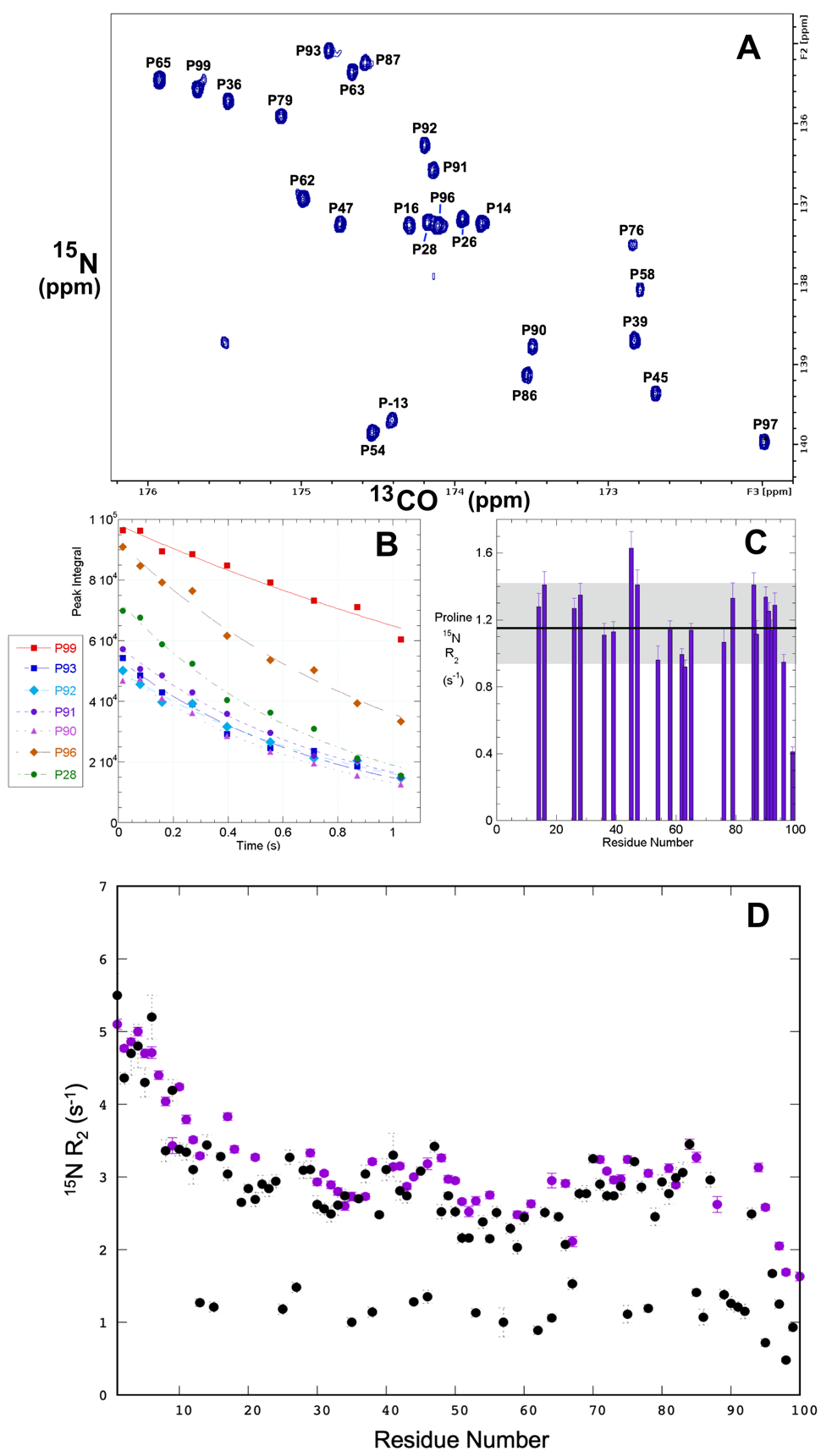


**A**. The ^13^C-detected ^15^N relaxation experiment obtained using a 15.9 ms delay. Proline ^15^N atoms are labeled. **B**. Fits of some representative proline ^15^N nuclei are shown Peaks “P-13”, which comes from the His tag, and P97, whose resonance is folded, were not included in the analysis. The R_2_ rates and their one standard deviation uncertainties of their fits (error bars) from the Topspin analysis are shown in **panel C**. NMRPipe analysis yielded similar results (data not shown). The black line marks the mean R_2_ rate (1.18 s^-1^) and the gray shaded area is the one standard deviation (+/- 0.24 s^-1^) from the mean of all the proline R_2_ rates. As previously observed in two recent studies (Murrali *et al.,* (2018); Mateos *et al.,* 2020), the R_2_ rates for the proline imine ^15^N are consistent with *trans* Xaa-Pro peptide bond (Mateos *et al.,* 2020) and are significantly slower relative those for the 19 amino acid residues. The residues near the C-terminus, especially P99, shows slower relaxation which is indicative of high flexibility (**panel C**). In contrast, the prolines of the two “PQP” mini-motifs (P14 & P16) and (P26 & P28) flanking the Q_4_RQ_4_ motif show slightly higher R_2_ rates as do P45 & P47 (which are found within a cluster of charged residues) and P90, P91, P92 & P93 (which adopt a short PPII helix). This is indicative of slightly less flexibility. Similar trends were observed recently in isolated Pro residues and a run of four consecuetive proline residues in the ID4 linker domain of the CREB binding protein (Murrali *et al.,* (2018). Finally the ^13^C-detected relaxation experiment was repeated for all hCPEB3 segment 1 residues using a broad sweep width and the results are shown in **panel D** as black circles along the results obtained using a ^15^N detected experiment, which are colored purple. The good agreement is a further confirmation of the presence of a rigid, partially populated α-helix at the N-terminus of Segment 1 (**Figure 2, Sup. Fig. 10 & 12**).

_____________________

Mateos B *et al.,* (2020) J. Mol. Biol. 432: 3093-3111. (ref. # 76 in the main text).

Murrali *et al.,* (2018) Chembiochem 19: 1625-1629 (ref. # 75 in the main text).

**Fig. S10** Partial Formation of an α-Helix in the N-terminal Residues of hCPEB3


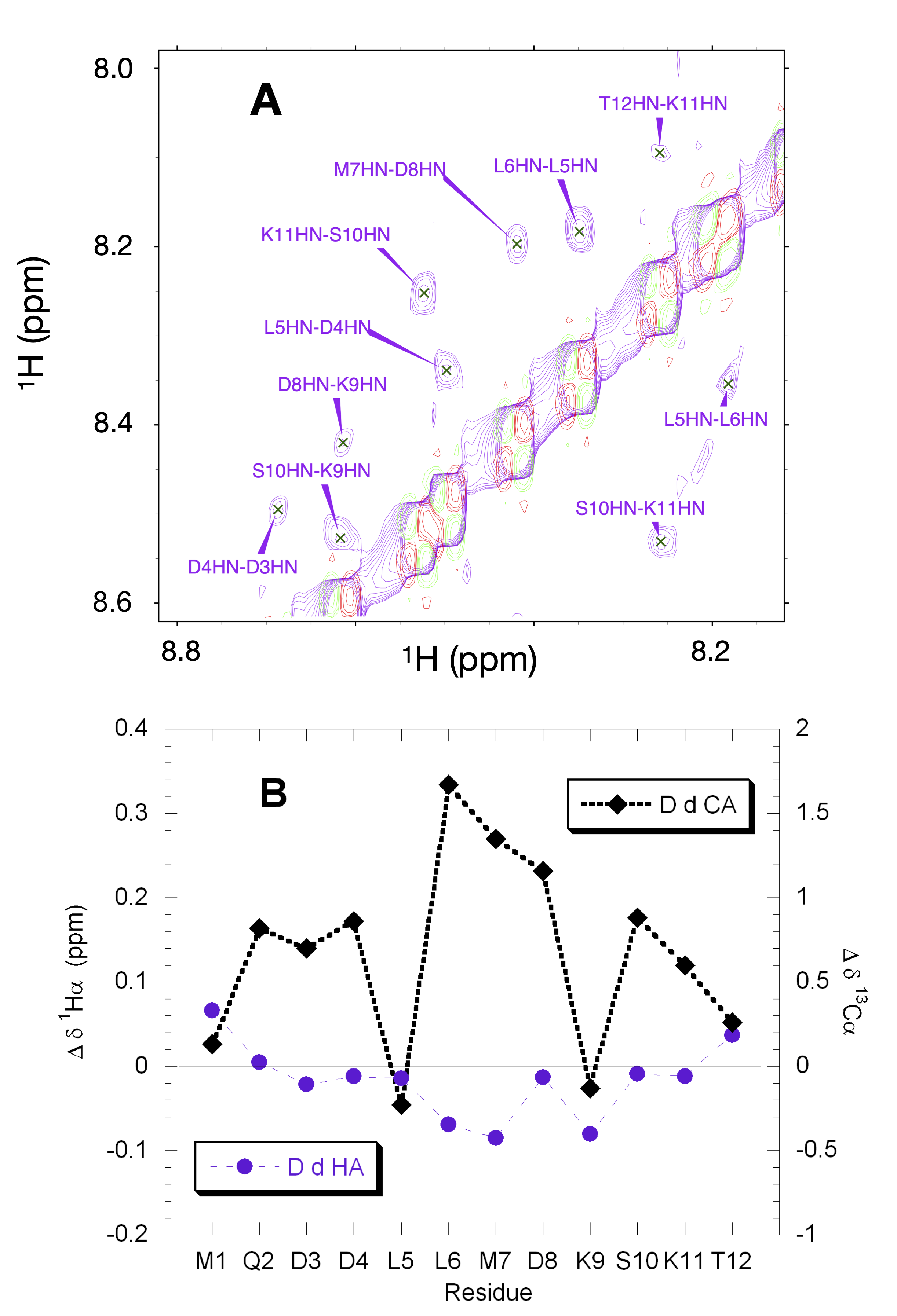


**A**. 2D ^1^H-^1^H NOESY (80 ms mixing time, **purple**) of a peptide corresponding to the initial residues of hCPEB3. Sequential NOE correlations, which are consistent with α-helical structure, are labeled. **Red** and **green** peaks along the diagonal are from the 2D ^1^H-^1^H COSY spectrum.

**B**. Conformational ^1^H (**purple**) and ^13^C (**black**) chemical shifts at 5 ºC for a peptide corresponding to the initial residues of hCPEB3 in 20% hexafluoroisopropanol (CF_3_-CHOH-CF_3_) The positive Δδ^13^C and negative Δδ ^1^H values are indicative of the formation of significant α-helix formation.

**Fig. S11** The Consecutive Proline Residues of hCPEB3 Show a Characteristic Pattern of Conformational Chemical Shifts and Weak Binding to Profilin.

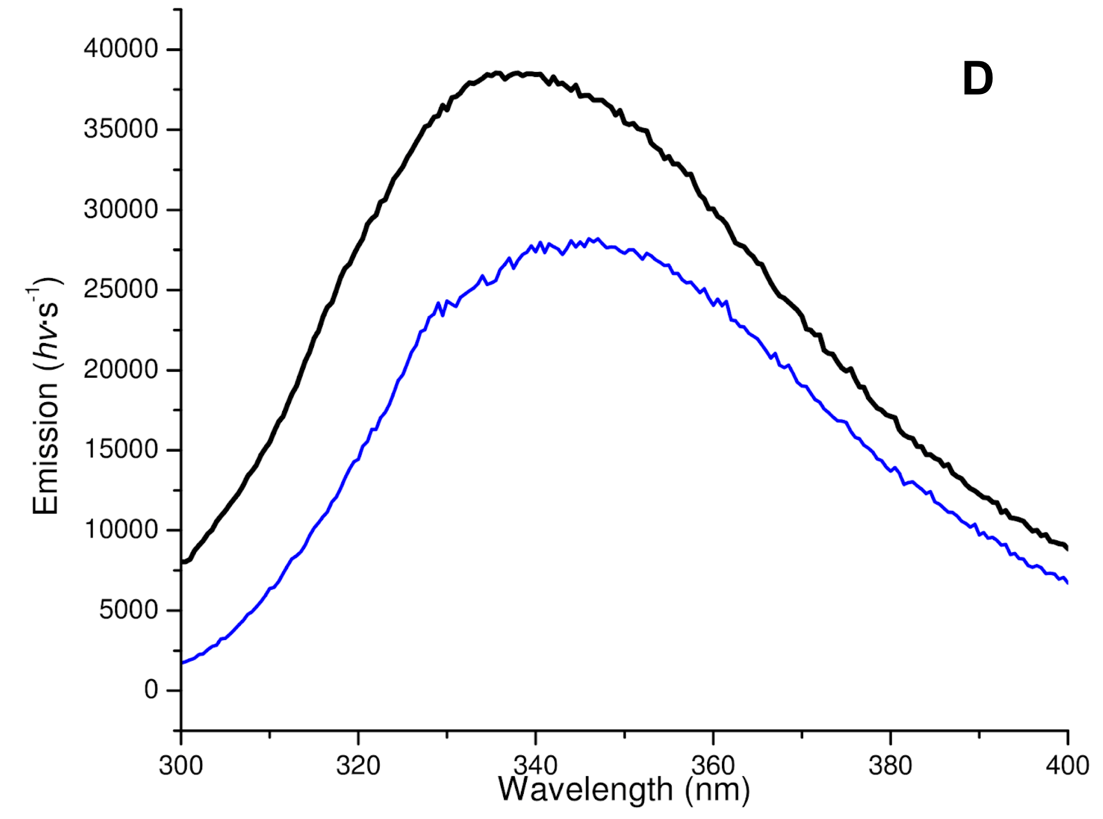


**A**. and **B**. Conformational chemical shifts (Δδ) for ^13^Cα (**blue** bars), ^13^Cβ (**red**) and ^13^CO (**green**) nuclei for residues in two Pro-rich stretches, H84-Q94 and Q165-175, each of which contains four consecutitive proline residues which will be locked in the polyproline II helical conformation. The Δδ values of residues P90, P91, P92, P168, P167 and P168 were averaged to obtain the values given in **Table 3** on the main text.

**C**. Isolated proline residues, *i.e.* those which do not have another proline residue within two anterior or posterior positions along the sequence, do not show a pattern of significant Δδ deviations.

**D**. Fluorescence emission spectra of 8.3 μM human profilin 1 in the absence (**blue** spectrum) or presence (**black**) of 4.5 mM acGPPPPAPAPQPam peptide at 20 ºC in 10 mM K_2_HPO_4_, 75 mM KCl, (pH 8.0) at 20 ºC. The enhanced, blue-shifted emission is indicative of binding [34].

**Fig. S12.** Residue Level Dynamics of hCPEB3’s Instrinsically Disordered Region


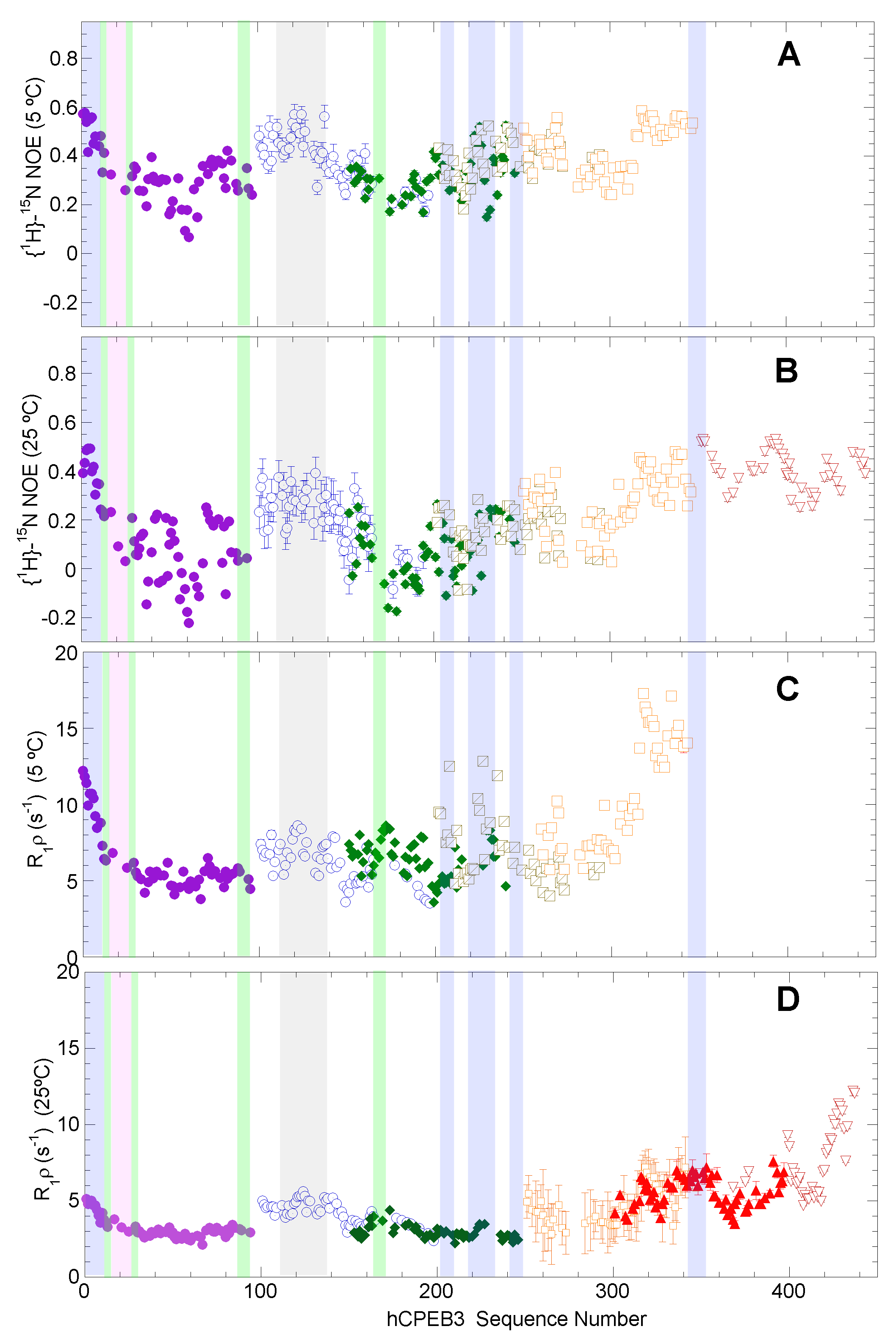


Residue-level dynamics on ns/ps time scales at 5ºC (**A**) and 25ºC (**B**) and on μs/ms at 5ºC (**C**) and 25 ºC (**D**). In panels **A** and **B**, a value of 0.86 is expected for completely rigid H-N groups; negative values are characteristic of a high flexibility. In panels **C** and **D**, while values less than 4 s^-1^ are hallmarks of flexibility, higher values mean decreased mobility. Data from individual segments are colored differently: segment 1 = **purple filled circles**, segment 3 = **blue open circles**, segment 4 = **green diamonds**, segment 5 = **brown barred open squares**, segment 6 = **orange open squares**, segment 7 = **filled red triangles**, segment 8 = **open inverted maroon triangles**. No data are represented for segments 7 and 8 at 5ºC, segment 5 in panel **D**, and segment 7 in panel **B** as the spectra obtained were of insufficient quality. The lack of other values is due to ^1^H^15^N signal overlap or proline residues whose nitrogen lacks a hydrogen. Error bars represent uncertainties as estimated from peak intensity signal/noise for panels **A** and **B** and as obtained from the fit of a signal exponential decay function to the peak intensity versus delay time data for panels **C** and **D**. The error bar is frequently smaller than the data symbol.

Elements of partial structure/interest are shaded **blue** for α-helices and **gray** for hydrophobic segments; these elements generally show decreased mobility. By contrast, the polyQ tract (shaded **magenta**) is more flexible. The dynamics of the PPII tracts (**green**) can not be assessed directly here as these imine residues lack ^1^HN. However, the dynamics of the PPII tract of segment 1 was analyzed by ^13^C-detected ^15^N-relaxation experiments and is shown in Sup. Fig. 9.

**Fig. S13.** ^1^HN-^1^Hα Coupling Constants for Segment 5

Confirm the Presence of α-Helices.

Intraresidual three bond ^1^HN-^1^Hα coupling constants (^3^J_1HN1Hα_) for segment 5 of hCPEB3. Regions with ^3^J_1HN1Hα_ < 5 (shaded **cyan**) correspond to two α-helices, in line with the results based on chemical shift deviations (*see* ***Fig. 3*** *in the Main Text*). By contrast, the last, modestly populated α-helix identified by chemical shift deviations (A238-Q246), does not show significantly lower ^3^J_1HN1Hα_ values. A zone with ^3^J_1HN1Hα_ > 7 Hz (shaded **rose**) indicates extended conformations. This zone spans the (VG)_5_ motif.

**Fig. S14**. Helices from Pathological and Functional Amyloids

Are Stabilized by Distinct Interactions.

Helical wheel diagrams of TDP-43, Aplysia CPEB (ApCPEB), Drosophila CPEB Orb2A and hCPEB3, nonpolar residues are colored **black**, aromatics=**purple**, Q/N=**magenta**, E/D=**red**, H/K/R=**blue**, G=g**reen**, C,S,T=**cyan**. The five hCPEB3 helices were identified here by NMR data. The TDP-43 helix, which is implicated in harmful amyloid formation is more hydrophobic and was previously identified by NMR by Lim *et al.,* 2016. The ApCPEB and Orb2A helices are putative as they have not yet been confirmed experimentally.

________________

Lim L, Wei Y, Lu Y, Song J. ALS-Causing Mutations Significantly Perturb the Self-Assembly and Interaction with Nucleic Acid of the Intrinsically Disordered Prion-Like Domain of TDP-43. PLoS Biol. 2016;14:e1002338.

**Fig. S15**.

Phosphorylation of S224 may increase the α-helix population of the S224-A233 segment.

The regions of ^1^H-^13^C HSQC spectra showing Ala ^13^Cα-^1^Hα (**panel A**) and ^13^Cβ-^1^Hβ (**panel B**) resonances of the peptide acEAVAAAAAAKKnh2, which corresponds to the helical segment S_224_AVAAAAAAAAA_235_ recorded at 5.0 ºC and pH* 3.22 (**red**) and pH* 5.34 (**blue**) are shown. pH* is the pH meter reading measured in D_2_O plus 0.40 pH units to account for the deuterium isotope effect. “ac” and “am” are terminal acetyl and amide moieties added to avoid end charges which would not be present in the hCPEB3 IDR. The substitution of the last two Ala (corresponding to A234 and A235 in the hCPEB3 sequence) for two Lys residues at the C-terminus of this segment was necessary to increase the peptide’s solubility as previous attempts to synthesize the wild type sequence led to insoluble products. Under these conditions, the N-terminal Glu residue is mostly charged at pH 5.34* and chiefly neutral at pH* 3.22, mimicking partially the effect of a phosphorylated or nonphosphorylated, respectively, Ser residue. It can be seen that the ^13^Cα chemical shift of Ala residue signals, which are overlapped, increases by about 0.15 ppm upon increasing the pH. The overlapped Ala ^13^Cβ signals decrease in chemical shift by approximately 0.07 ppm. These chemical shift changes correspond to an increase of 5 - 10 % in α-helicity based on an increase of 3.1 ppm for ^13^Cα and a decrease of 0.76 ppm for ^13^Cβ expected for 100% α-helix formation, therefore providing support for helicity increasing upon phosphorylation.

**Fig. S16.**

Insight into interhelix interactions from Förster resonance energy transfer

Fluorescence spectra of two polypeptides whose concentration was 10.0 μM: MQDDLLMDKSKTGGGGASSSWNTHQ  called “Trp”

and Dansyl-MQDDLLMDKSKTGGGGASSSWNTHQ   called “Dan-Trp”, with residues corresponding to the first and fourth partly populated α-helices of hCPEB3 (shaded pink and cyan, respectively) were recorded.

It can be seen in the graph on the *left* (in buffer) that the emission of Trp is high in “Trp” but low in the “Dan-Trp” peptide.  This is due to energy transfer of Trp to Dan and thanks to this the Dansyl shines and there is an emission centered at 560 nm which is not seen for the “Trp” peptide.

Applying the theory of Förster and making usual assumptions about the rapid movement and averaged orientation of the donor and acceptor groups, and using the known “Förster distance” (R_0_) of 23.6 angstroms Lakowicz *et al.* (1990), the mean distance between the Dansyl and Trp can be calculated to be 22 angstroms, which is significantly lower than the mean value (41 angstroms) expected for a statistical coil of 21 residues (the number of residues separating the Dansyl and Trp groups) as calculated on the basis of the data complied by Fitzkee and Rose (2004) PNAS·USA **101**(34):12497-12502 as well as the results reported by Tanford *et al.* (1966) J. Biol. Chem. **241**(8) 1921-1923.


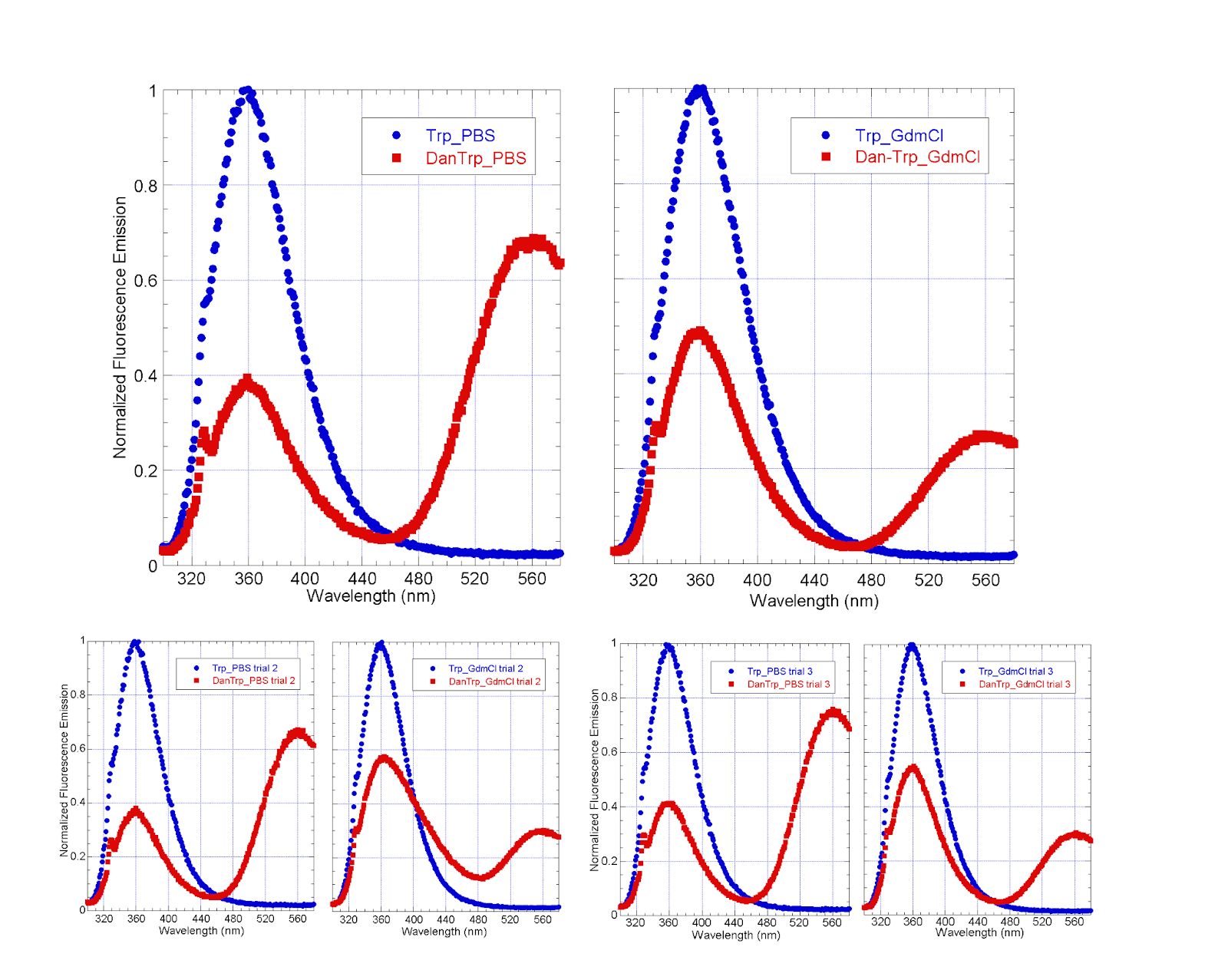


On the other hand, in the presence of 7.4 M GdmCl, in the graphs shown on the *right*, the Trp emission intensity decrease due to Dansyl is smaller, and also the emission intensity of Dansyl is less intense. Two repetitions of the experiment are shown in the lower panels. Based on these results, one can conclude that the Trp and Dansyl groups are farther away in the presence of the denaturant, due to the unfolding of a more compact structure that is present in the absence of GdmCl.  Thus, these new results support the possibility of interhelical contacts in hCPEB3.
